# DrugSim2DR: systematic prediction of drug functional similarities in the context of specific disease for drug repurposing

**DOI:** 10.1093/gigascience/giad104

**Published:** 2023-12-19

**Authors:** Jiashuo Wu, Ji Li, Yalan He, Junling Huang, Xilong Zhao, Bingyue Pan, Yahui Wang, Liang Cheng, Junwei Han

**Affiliations:** College of Bioinformatics Science and Technology, Harbin Medical University, Harbin 150081, China; College of Bioinformatics Science and Technology, Harbin Medical University, Harbin 150081, China; College of Bioinformatics Science and Technology, Harbin Medical University, Harbin 150081, China; College of Bioinformatics Science and Technology, Harbin Medical University, Harbin 150081, China; College of Bioinformatics Science and Technology, Harbin Medical University, Harbin 150081, China; College of Bioinformatics Science and Technology, Harbin Medical University, Harbin 150081, China; College of Bioinformatics Science and Technology, Harbin Medical University, Harbin 150081, China; College of Bioinformatics Science and Technology, Harbin Medical University, Harbin 150081, China; College of Bioinformatics Science and Technology, Harbin Medical University, Harbin 150081, China

**Keywords:** computational drug repurposing, drug–drug similarity, network analysis, specific disease state

## Abstract

**Background:**

Traditional approaches to drug development are costly and involve high risks. The drug repurposing approach can be a valuable alternative to traditional approaches and has therefore received considerable attention in recent years.

**Findings:**

Herein, we develop a previously undescribed computational approach, called DrugSim2DR, which uses a network diffusion algorithm to identify candidate anticancer drugs based on a drug functional similarity network. The innovation of the approach lies in the drug–drug functional similarity network constructed in a manner that implicitly links drugs through their common biological functions in the context of a specific disease state, as the similarity relationships based on general states (e.g., network proximity or Jaccard index of drug targets) ignore disease-specific molecular characteristics. The drug functional similarity network may provide a reference for prediction of drug combinations. We describe and validate the DrugSim2DR approach through analysis of data on breast cancer and lung cancer. DrugSim2DR identified some US Food and Drug Administration–approved anticancer drugs, as well as some candidate drugs validated by previous studies in the literature. Moreover, DrugSim2DR showed excellent predictive performance, as evidenced by receiver operating characteristic analysis and multiapproach comparisons in various cancer datasets.

**Conclusions:**

DrugSim2DR could accurately assess drug–drug functional similarity within a specific disease context and may more effectively prioritize disease candidate drugs. To increase the usability of our approach, we have developed an R-based software package, DrugSim2DR, which is freely available on CRAN (https://CRAN.R-project.org/package=DrugSim2DR).

## Background

Cancer is one of the most common complex diseases in terms of morbidity and mortality, and because of the complexity and diversity of cancer, finding effective therapeutic drugs for patients with cancer remains a formidable challenge. At present, there is widespread interest among researchers in drug repurposing methods. Drug repurposing is defined as the process of applying an existing drug ingredient to a new indication [1, 2]. Compared to traditional drug discovery, drug repurposing has higher efficiency and lower risk due to a more comprehensive understanding of the safety and toxicity profiles of existing drugs [3]. In addition, since the drug has already gone through clinical trials, it has a higher probability of being approved for use in the clinic [4]. In recent years, researchers have focused on drug repurposing *in silico* approaches [5]. With advances in sequencing technology and the availability of large amounts of molecular omics data, there is a better prospect of systematically inferring the new relationship between drugs and/or diseases [1], which will facilitate the development of computational drug repurposing approaches.

Several computational techniques have been documented for drug repurposing, drawing upon expertise in bioinformatics and systems biology. For example, Connectivity Map (CMap) is a groundbreaking algorithm proposed by Lamb et al. in 2006 [6]. Through in-depth analysis of the reverse association between drug-induced and disease-induced gene expression profiles, CMap unveils valuable insights into the underlying mechanisms and offers new avenues for drug repurposing. Subsequently, Drug versus Disease (DvD) [7] improves the CMap approach and performs drug repurposing by comparing drug and disease gene-based signatures and evaluating their reverse relationship. SubtypeDrug identifies abnormal subpathways induced by diseases and drugs, respectively, and then evaluates the reverse correlation between drugs and diseases at the subpathway level for drug repurposing [8, 9]. DRviaSPCN repurposes drugs for cancer by considering drug-induced subpathways and their crosstalk effect [5]. Such approaches generally perform drug repurposing by evaluating drug–disease reverse association at the gene expression or pathway activity level. Although these approaches effectively discover some cancer candidate drugs, they do not incorporate the similarities/interactions between drugs, which is a research area that has to be thoroughly explored [10]. PriorCD is a network-based drug repurposing approach that constructs a drug functional similarity network at the pathway level and uses a global network propagation algorithm to prioritize candidate cancer drugs [11]. Groza et al. [12] constructed a drug similarity network based on the drug–target interactions for drug repurposing. PIMD predicts novel therapeutic uses of drugs based on a drug similarity network constructed by integrating chemical, pharmacological, and clinical data of drugs [13]. These network-based approaches achieved good results for drug repurposing; however, they generally constructed drug–drug similarity/interaction networks based on drug targets, chemical structures, semantic similarity, and so on and neglected disease-specific molecular characteristics. Indeed, complex diseases, especially cancer, are highly heterogeneous, and different cancers exhibit varying molecular characteristics and biological processes during their development [14]. In different diseases, the drug similarities/interactions will be diverse. Therefore, it is indispensable to incorporate molecular characteristics in the context of a specific disease state for inferring drug–drug relationships and drug repurposing.

In this study, we presented a novel network-based approach, named DrugSim2DR, for drug repurposing based on a weighted drug–drug functional similarity network constructed with the transcriptionally altered genes in the context of the specific disease state. In the approach, we first constructed the drug–drug similarity network with the gene expression profiles between a pair of binary conditions (e.g., case/control, normal/diseased). In the drug–drug similarity network, the links and their weights reflect both the extent to which the targets of drugs shared biological function and the differential transcriptional level of target genes common to those drugs. Drugs were prioritized according to the network centrality scores calculated by a network propagation algorithm, and then a bootstrap-based approach was used to estimate the statistical significance of the drug centrality scores. We applied DrugSim2DR to datasets of breast cancer and lung cancer and achieved better predictive performance that surpassed some other classical drug repurposing approaches, demonstrating the potential of our approach as a practical drug repurposing tool. We also developed a tool package called “DrugSim2DR” for implementing our approach, which is freely available from https://CRAN.R-project.org/package=DrugSim2DR.

## Materials and Methods

### Data acquisition and processing

To construct a drug similarity network and perform the drug repurposing, we collected 5,804 drugs with corresponding targets from the DrugBank database [15]. We selectively retained drugs possessing a target gene count of over 3 but fewer than 500 and obtained 1,289 drugs. This screening strategy is deemed appropriate as it avoids the introduction of network bias and ensures maximal inclusion of drug data. Then, Molecular Function (MF) gene sets derived from Gene Ontology (GO) were downloaded from the Molecular Signature Database [16]. We believe that employing MF gene sets from the GO database allows us to attain a heightened precision in comprehending the interplay between drugs and biomolecules, consequently unveiling the mechanisms of drug action. This is crucial for comprehending the functions of drugs and for accurately assessing the similarity between them. We retained MF sets with gene numbers greater than 5 and less than 100, resulting in a total of 1,330 MF sets. This will avoid overly narrow or broad functional sets. To comprehensively assess and comprehend our methodology, we applied our approach to multiple types of cancer datasets. The transcriptional data of different cancer types were downloaded from the Gene Expression Omnibus (GEO) database [17], including breast cancer (GSE53752 [18], GSE42568 [19], and GSE21422 [20]), lung adenocarcinoma (GSE68465 [21], GSE31210 [22], and GSE74706 [23]), T-cell prolymphocytic leukemia (GSE5788 [24]), renal cell carcinoma (GSE53757 [25]), and head and neck squamous carcinoma (GSE6631 [26]). Each set of gene expression data used contained nonpaired disease and control samples. In these datasets, the expression values for each gene were standardized to a normal distribution using the *z*-score normalization method across all samples.

### Calculating transcriptional dysregulation levels of genes

**Figure 1:**
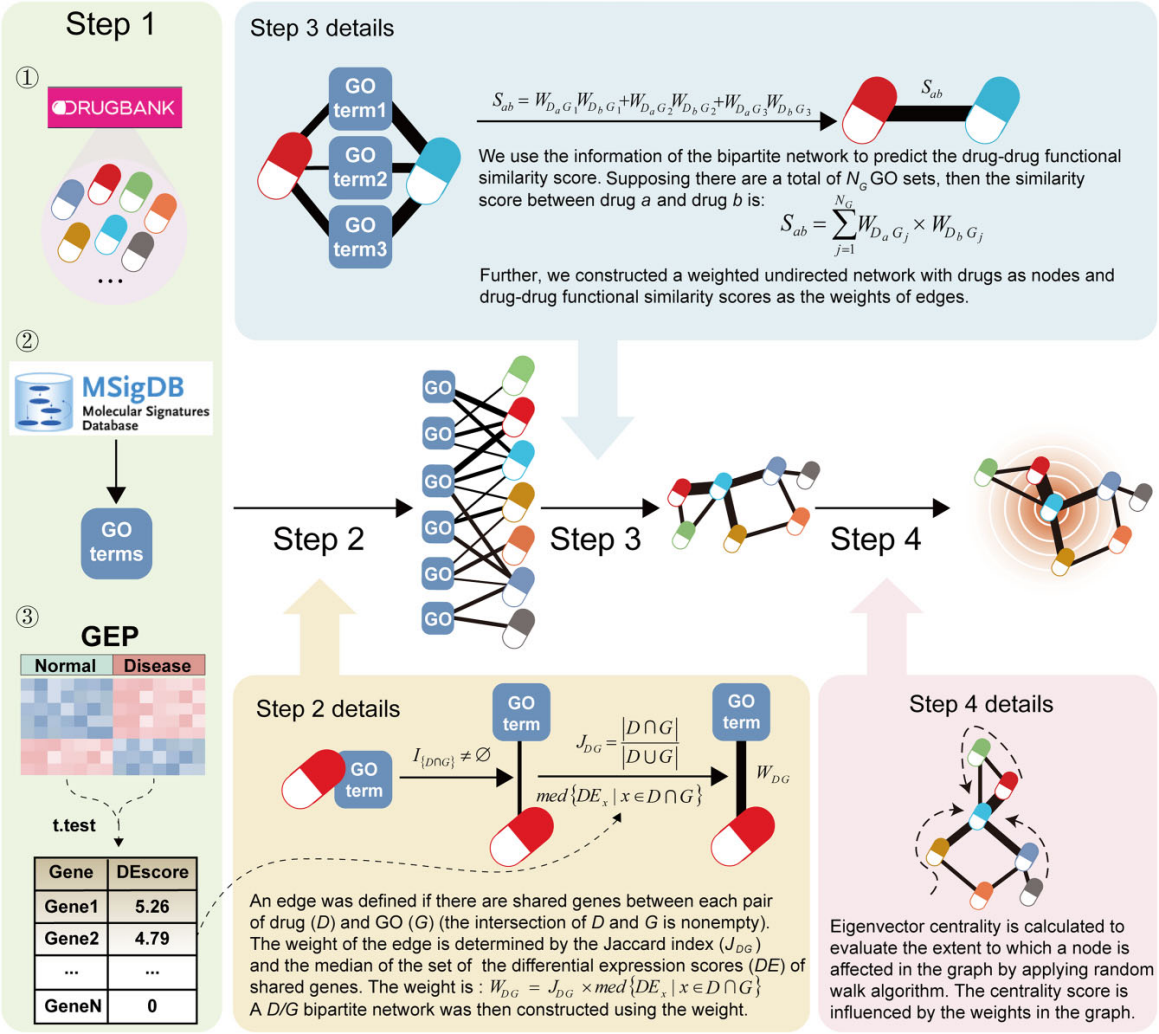
Schematic overview of the DrugSim2DR method.

We estimated the transcriptional dysregulation level of genes in the context of a specific disease (step 1 in Fig. 1). For each gene, the 2-tailed *t*-test was applied to assess the gene differential expression extent between normal and disease samples. Then, we transformed the *P* value of the *t*-test of each gene to *z*-score through *z* = φ^−1^(1 − p), where φ^−1^ is the inverse normal cumulative density function. We defined the absolute value of the *z*-score as the differential expression (DE) score to reflect the extent to which gene expression is affected by the disease. The higher the DE score, the stronger the gene is transcriptionally dysregulated.

### Constructing a *Drug/GO* bipartite network under the disease context

In step 2, we intended to build a bipartite network using drugs and GO gene sets (terms) as nodes (step 2 in Fig. 1). If the targets of a drug exist in a GO term, we believe that the GO term is related to this drug. We quantified the relationship between a drug (*D*) and a GO term (*G*) by a weighted undirected edge. The weight (*W_DG_*) was defined as follows:


(1) \begin{eqnarray*}
{J}_{DG} = \frac{{\left| {D \cap G} \right|}}{{\left| {D \cup G} \right|}}
\end{eqnarray*}

(2) \begin{eqnarray*}
{W}_{DG} = {J}_{DG} \times med\left\{ {D{E}_x|x \in D \cap G} \right\}
\end{eqnarray*}

*J_DG_* is the Jaccard index, which was used to measure the degree of overlap between a GO term and a target gene set for a drug. We believe that the relationship between drugs and GO terms is decided not only by the overlap but also by the transcriptional dysregulation of the overlapped genes in the disease context. Thus, we calculated *med*{*DE_x_*| *x*∈*D*∩*G*}, the median DE score of all genes common to targets of a drug and a GO term, which reflects the degree of transcriptional dysregulation in the disease state of overlapped genes. Finally, with *W_DG_* as the weight of the edge between *D* and *G*, reflecting the disease-modulated *D/G* relationship, a *D/G* bipartite network was constructed. The final network is represented by an adjacency matrix *W*, in which rows are drugs, columns are GO terms, and elements represent the degree of association between drugs and GO terms.

### Constructing a drug–drug functional similarity network

#### Calculating drug–drug functional similarity scores

In step 3, we first used the information from the *D/G* bipartite network to calculate the functional similarity score between 2 drugs (step 3 in Fig. 1). We consider that the functional similarity score between 2 drugs will be stronger when they share more neighbor GO terms. Thus, we define the similarity score between a pair of drugs (*D_a_* and *D_b_*) as follows:


(3) \begin{eqnarray*}
{S}_{ab} = \mathop \sum \limits_{j = 1}^{{N}_G} {W}_{{D}_a{G}_j} \times {W}_{{G}_j{D}_b}
\end{eqnarray*}

where *N_G_* is the total number of shared GO terms between the 2 drugs, and *W_DaGj_* or *W_DbGj_* is the edge weight between *D_a_* or *D_b_* and GO term *G_j_*. That means the functional similarity score between *D_a_* and *D_b_* is the sum of the contributions concerning the disease-induced transcriptional dysregulation of all GO terms shared between them.

#### Constructing a drug–drug functional similarity network based on the D/G bipartite network

Furthermore, we constructed a drug–drug functional similarity network, in which the edge weights represent the similarity scores between drugs. To represent the drug–drug network, we defined an adjacency matrix *A*:


(4) \begin{eqnarray*}
A = W \cdot {W}^T
\end{eqnarray*}

where *W* is the adjacency matrix of the *D/G* bipartite network. Thus, the elements of *A* correspond to the functional similarity scores (see equation (3)) between drugs in the drug–drug similarity network. We assign the value of 0 to the diagonal element of matrix *A*, indicating self-links were removed. After these steps, a drug functional similarity network under a specific disease context is then constructed, which comprises a total of 1,289 drug nodes and 322,250 edges. This process was previously used to construct a cell–cell crosstalk network [27].

### Prioritizing disease candidate drugs through a network propagation algorithm

In the drug–drug functional similarity network, the edge weight reflects both the drug functional similarity and the disease-induced transcriptional dysregulation. It is reasonable to assume that the evidence that a drug may potentially treat a disease could be reinforced by the evidence of its neighbors. Thus, a drug is more inclined to act on a disease if it is linked to more neighbor nodes and the edges have larger weight. In step 4, we applied a network propagation algorithm, the random walk with restart, to calculate the eigenvector centrality score of drugs, which is a measure to determine the significance of disease candidate drugs. In this algorithm, the more central a drug node is, the more probable it is to be visited by the random walker and result in a larger eigenvector centrality score. To implement this algorithm, we defined a probability transition matrix *T* by col-normalizing the adjacency matrix *A*. The formula is as follows:


(5) \begin{eqnarray*}
{T}_{ab} = \frac{{{A}_{ab}}}{{\mathop \sum \nolimits_{a = 1}^{{N}_D} {A}_{ab}}}
\end{eqnarray*}

where *N_D_* is the total number of drugs in the network, and *T_ab_* denotes the probability of transferring from *D_a_* to *D_b_*. The formula of random walk with restart algorithm is as follows:


(6) \begin{eqnarray*}
{v}^{t + 1} = \left( {1 - r} \right)T{v}^t + r{v}^0
\end{eqnarray*}

where *T* is the probability transition matrix, and *r* is the restart probability. It has been demonstrated to have only a slight effect on the results when it fluctuates between 0.1 and 0.9 [25], so we set *r* = 0.9 in the study. *v*^0^ is the initial probability vector. The random walk with restart applied here is characterized as the limiting distribution resulting from a prolonged random walk. Due to the algorithm’s protracted runtime, it eventually becomes independent of the initial seed nodes, rendering the selection of starting seeds inconsequential. What matters lies in the strategic implementation of random restarts, particularly in networks featuring a robust community structure. And thus the seed nodes are selected from a uniform distribution in our study. To do this, the initial probability vector is constructed by assigning to each node the same value and making their sum 1.

The parameter *v^t^* is the probability vector comprising the probabilities of the nodes at step *t*. After a limited number of iterations, the probability vector *v^t^* will converge to a stable state *v* (the difference between *v^t^* and *v^t+1^* is less than 1e-10). Its *i*th element *v_i_* in the vector represents the eigenvector centrality score of drug *D_i_*. A larger eigenvector centrality score of a drug suggests a potential therapeutic effect on the disease.

To evaluate the statistical significance (*P* value) of the drug centrality score, we performed the permutation test. The drug centrality score was calculated in the drug similarity network, which was constructed based on the intersection of each pair of drug target gene sets and GO terms and their transcriptional dysregulation scores, DE scores. As the intersection is constant and the DE scores of genes are variable, we used bootstrap resampling of our original DE scores at the gene level and repeated steps 2 to 4 to find statistically significant candidate drugs. This produced a vector of random drug centrality scores, v*. By repeating this progress 1,000 times, a set of randomly generated drug centrality score vectors was produced, $\{ {{v}^{*1}, \cdots ,{v}^{*1000}} \}$. For given drug *D_i_*, we compared its original drug centrality score ${v}_i\ $ with the set of random scores $\{ {v_i^{*k}} \}_{k = 1}^{1000}$. And the *P* value of drug *D_i_* was calculated as follows:


(7) \begin{eqnarray*}
p - value\ \left( {{D}_i} \right) = \frac{{\mathop \sum \nolimits_{k = 1}^{1000} I\ \left\{ {{v}_i^{*k} \ge {v}_i} \right\}}}{{1000}}
\end{eqnarray*}

where *I* is the indicator function. To correct multiple comparisons, the *P* values were then adjusted using Benjamin and Hochberg’s false discovery rate (FDR) method. We have developed an available CRAN package called “DrugSim2DR” (RRID: SCR_024564) for implementing our approach.

## Results

### Case study: breast cancer

#### Calculating the drug–drug functional similarity scores in breast cancer

**Figure 2:**
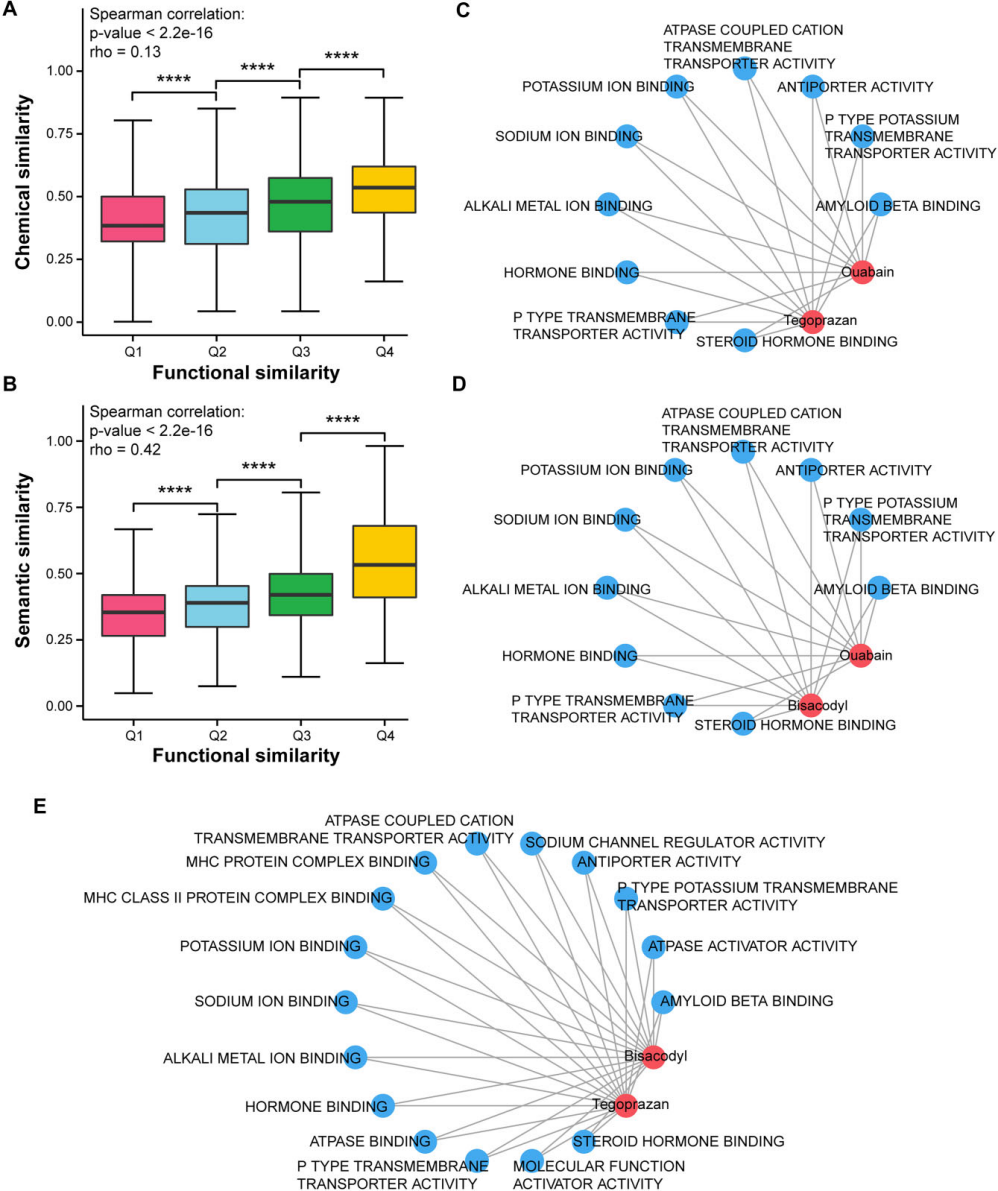
Assessment of drug–drug functional similarity in the context of breast cancer. (A, B) Comparations of chemical structure similarity and semantic-based similarity across 4 groups (Q1–Q4) were categorized according to the quartiles of the functional similarity scores. Statistical significance between boxplots is calculated by Wilcoxon rank-sum tests (^****^P < 0.0001). (C–E) Bipartite networks of drugs and their shared GO terms for ouabain/tegoprazan, ouabain/bisacodyl, and bisacodyl/tegoprazan pairs. The red nodes in the network represent drugs and the blue ones represent GO terms.

The study of drug–drug similarity has been used repeatedly in drug repurposing approaches. The current drug–drug similarity prediction approaches mainly depend on chemical structure- and semantic-based similarity. However, these approaches did not adopt the molecular features of disease, and the drug–drug similarity may be varied in different diseases. Our approach innovatively considers transcriptional dysregulation in the context of a specific disease. Using drug target gene sets, GO terms (Molecular Function), and the breast cancer gene expression profiles (GSE53752), we calculated the functional similarity score between each pair of drugs reflecting the extent to which incident drug action produces similar biological outcomes and the differential transcriptional activity of genes common to those drugs (see Materials and Methods). To test if our drug–drug functional similarity was associated with the traditional chemical structure- or semantic-based similarity (Supplementary Text 1), we categorized all drug pairs into 4 groups (Q1–Q4) according to the quartiles of our functional similarity scores. We found that our drug–drug functional similarities were significantly correlated with chemical structure similarities (Spearman’s rank correlation test, *P* < 2.2e-16) and semantic similarities (Spearman’s rank correlation test, *P* < 2.2e-16), respectively, and the drug pairs in the high functional similarity group have larger chemical structure similarities and semantic similarities compared with that of the low functional similarity group (Fig. 2A, B). The results demonstrated that our drug–drug functional similarity algorithm could identify biologically significant drug pairs.

To explain our results in detail, we highlighted the top 10 drug pairs with the highest similarity scores (Table 1). Notably, we discovered that some of these drug pairs also have larger chemical structure- and semantic-based similarities, such as fomepizole/pyrazole, benzthiazide/ellagic acid, and benzthiazide/cyclothiazide (Supplementary Fig. S1A, B). These findings are in line with our expectations. Moreover, as our drug–-drug functional similarity scores were calculated in the transcriptional profiling of breast cancer, we further tested if the scores could provide new insight into drug–drug similarity. Interestingly, we observed that 3 drug pairs (ouabain/tegoprazan, ouabain/bisacodyl, and bisacodyl/tegoprazan) had comparatively lower chemical structure and semantic similarities (Supplementary Fig. S1A, B) but showed large functional similarities in our algorithm (Table 1). This may be because they shared the same functional terms in the context of the breast cancer dataset. For example, ouabain and tegoprazan shared the same functional terms, including “Hormone binding” and “Steroid-hormone binding” (Fig. 2C). Moreover, most of these functional terms are associated with the development of breast cancer, which may result in their functional similarity. Specifically, several studies have shown that some estrogens or progestins can exacerbate the proliferation of breast cancer cells, and targeting hormone receptors is a widely used and effective treatment strategy [28, 29]. Furthermore, the drug pairs for ouabain/bisacodyl and bisacodyl/tegoprazan showed similar results (Supplementary Fig. S1A, B and Fig. 2D, E). This indicates that our approach may identify new functional similar drug pairs in the context of a specific disease, which may provide a new reference for prediction of drug combinations.

**Table 1:** Top 10 functionally similar drug pairs in the context of breast cancer

Drug 1	Drug 2	Drug name 1	Drug name 2	Similarity score
DB01213	DB02721	Fomepizole	4-Iodopyrazole	17.27
DB02721	DB02757	4-Iodopyrazole	Pyrazole	5.75
DB01213	DB02757	Fomepizole	Pyrazole	5.13
DB00562	DB00819	Benzthiazide	Acetazolamide	4.18
DB01092	DB16690	Ouabain	Tegoprazan	4.09
DB01092	DB09020	Ouabain	Bisacodyl	3.80
DB00562	DB08846	Benzthiazide	Ellagic acid	3.52
DB00562	DB00606	Benzthiazide	Cyclothiazide	3.46
DB09020	DB16690	Bisacodyl	Tegoprazan	3.32
DB00819	DB08846	Acetazolamide	Ellagic acid	3.31

#### Drug repurposing for breast cancer

**Figure 3:**
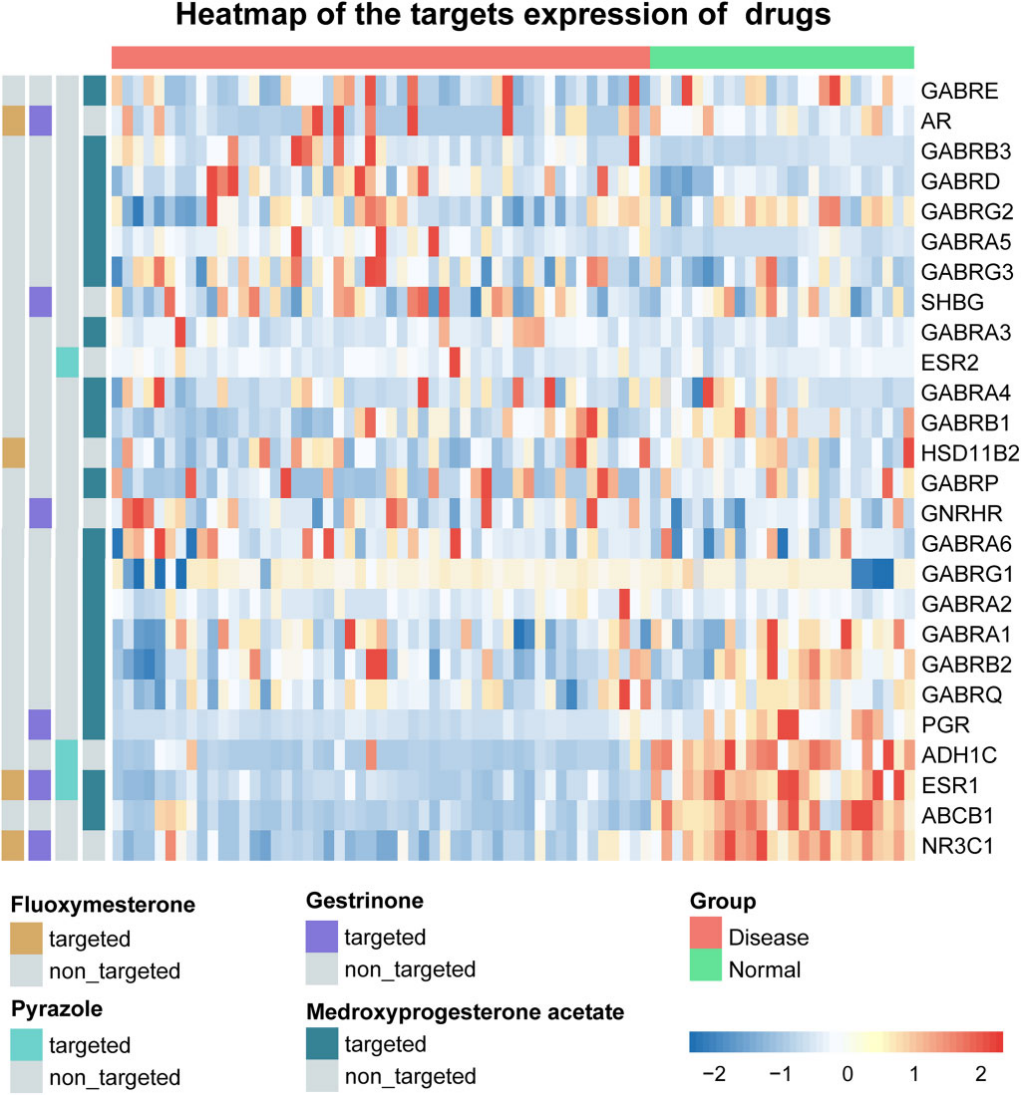
Heatmap of gene expression levels of drugs’ targets between breast cancer and normal samples.

Furthermore, by leveraging the functional similarity scores of all drug pairs in the context of breast cancer, we constructed a weighted drug–drug similarity network. Within this network, drug nodes are interconnected by edges with weights that directly correspond to the level of their functional similarity. A drug(s) that may potentially treat the disease can be expected to be reinforced by the evidence of its neighbors and their linked weights. The random walk algorithm with restart was then applied to the network to calculate the eigenvector centrality scores of drugs. Drug nodes are more central the more likely they are to be visited in the random walk and the larger the centrality scores they obtain. The significance of these centrality scores of drugs was assessed using a permutation test. With the default FDR <0.1, our DrugSim2DR approach identified 5 potential anti–breast cancer drugs (Table 2). An interesting observation is that fluoxymesterone, the top-ranked drug, has received US Food and Drug Administration (FDA) approval for breast cancer treatment. Fluoxymesterone could competitively block estrogen receptors, preventing the development of hormone-dependent tumor lines [30, 31]. Moreover, we also identified some potential candidate drugs with evidence that they may inhibit the development of breast cancer. For example, gestrinone has been demonstrated to have anticancer effects in cell experiments, particularly in the field of gynecological cancer [28]. Pyrazole is a heterocyclic organic compound, and the effect of its derivative on breast cancer has been confirmed by multiple studies. Ashourpour et al. [32] provided evidence that pyrazole derivatives can cause apoptosis in MDA-MB-468 cells (breast cancer cell line) through reactive oxygen species. Gutierrez et al. [33] proved that a pyrazole-based derivative P3C has demonstrated resistance to breast cancer, meaning a great anticancer therapy. Additionally, medroxyprogesterone acetate acts as a progestin receptor agonist and suppresses the proliferation of cancer cells in estrogen receptor–positive breast cancer [34]. To further elucidate the therapeutic potential of these candidate drugs, we tested the expression levels of target genes of the 4 candidate drugs between breast cancer and normal samples, and they exhibited contrasting expression differences (Fig. 3). This suggests that these drugs may intervene in the disease process by regulating target expression and may become meaningful treatment options.

**Table 2:** Candidate drugs for breast cancer identified by DrugSim2DR with FDR <0.1

DrugBank ID	Drug name	Main indications	Centrality score	FDR	Evidence
DB01185	Fluoxymesterone	Breast cancer/hypogonadism	0.0051	<0.001	FDA approved
DB11619	Gestrinone	Endometriosis	0.0050	<0.001	PMID: 34921996
DB02757	Pyrazole		0.0045	<0.001	PMID: 34319030
DB01213	Fomepizole	Ethylene glycol poisoning	0.0026	<0.001	
DB00603	Medroxyprogesterone acetate	Metastatic renal cell carcinoma/metastatic endometrial carcinoma	0.0025	<0.001	PMID: 31805393

### Case study: lung cancer

We then used a lung cancer dataset (GSE68465) to illustrate the effectiveness of DrugSim2DR in prioritizing cancer candidate drugs. With FDR <0.1, DrugSim2DR identified 9 potential anti–lung cancer drugs (Supplementary Table S1). Interestingly, we identified 2 FDA-approved drugs for the treatment of lung cancer, which are methotrexate and pemetrexed. Moreover, some candidate drugs with positive evidence have also been identified. For example, seliciclib is a potent cyclin-dependent kinase inhibitor that is currently undergoing phase 2 clinical testing in lung and B-cell malignancies. Seliciclib has been shown to induce cell cycle arrest and apoptosis in cancer cells [35]. Olomoucine is a cyclin-dependent kinase inhibitor. Some available studies have confirmed that olomoucine can inhibit the G1/S transition of cells and thus inhibit the proliferation of cancer cells [36, 37]. SU9516 is a cyclin-dependent kinase 2 inhibitor, exhibiting an inhibitory effect on the epithelial–mesenchymal transition (EMT) in A549 lung cancer cells. Specifically in tumor cell emergence and migration, EMT is a significant pathogenic process in cancer [38]. This ability to inhibit EMT predicts the potential of SU9516 as a lung cancer drug candidate. Alvocidib effectively inhibits EML4-ALK cells, driving lung cancer progression, thereby suppressing tumor growth and inducing apoptosis [39]. Alvocidib and docetaxel are in phase 2 clinical trials for the treatment of non–small cells [40]. We also tested the expression levels of target genes of the 7 candidate drugs between lung cancer and normal samples, and they exhibited contrasting expression differences (Supplementary Fig. S2).

These results indicate that DrugSim2DR can offer precise and varied treatment choices to patients. It can expedite drug discovery and furnish novel prospects and opportunities for subsequent research and clinical application.

### Evaluating the DrugSim2DR approach

#### Performance of the DrugSim2DR approach

**Figure 4:**
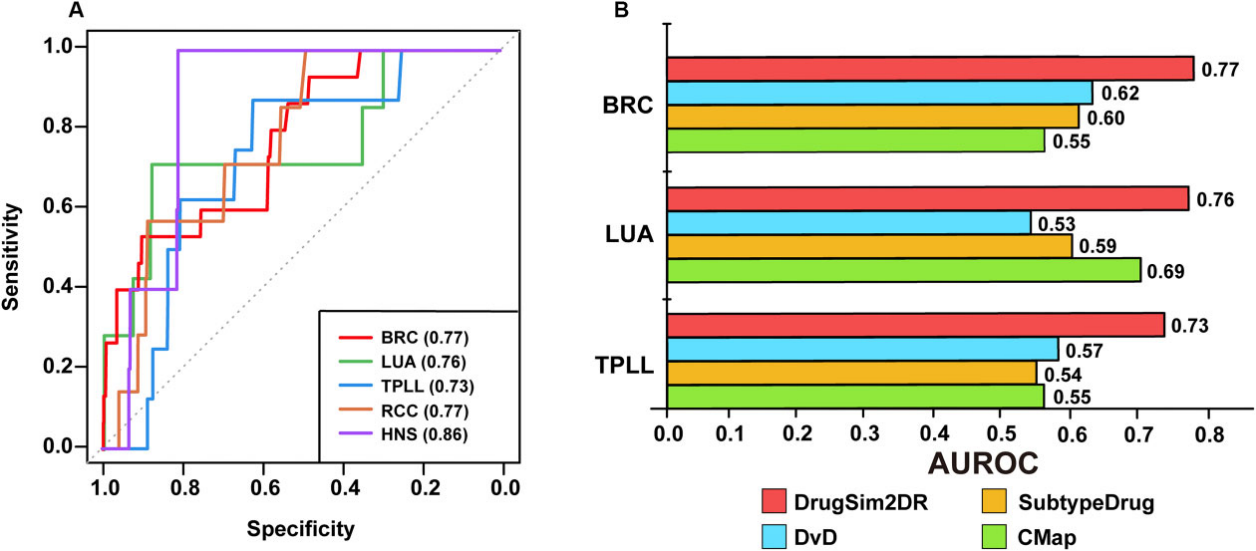
Performance of the DrugSim2DR approach. (A) ROC curves for drugs identified by DrugSim2DR in 5 different cancer types. The AUROC values for drugs in each cancer type are calculated and displayed respectively. (B) Comparison of DrugSim2DR with 3 other approaches. We apply DrugSim2DR to 3 cancer types to compare the performance with the CMap, SubtypeDrug, and DvD. AUROC values are used to compare their performance.

To evaluate the reliability of the DrugSim2DR approach, we performed the receiver operating characteristic (ROC) curve analysis according to the centrality scores of drugs. The FDA-approved drugs for the specific cancer types were collected from the DrugBank database [15] and used as the true-positive drug set. In the above breast cancer (BRC) and lung cancer (LUA) datasets, the values of area under the ROC curve (AUROC) reached 0.77 and 0.76, respectively (Fig. 4A). To confirm the accuracy and wide applicability of DrugSim2DR more comprehensively, we then applied it to 3 other cancer gene expression datasets: T-cell prolymphocytic leukemia (TPLL), renal cell carcinoma (RCC), and head and neck squamous carcinoma (HNS) (see Materials and Methods). As shown in Fig. 4A, the values of AUROC of our approach were 0.73 for TPLL, 0.77 for RCC, and 0.86 for HNS. These results indicate that DrugSim2DR could effectively identify cancer candidate drugs.

#### Comparison of DrugSim2DR with other approaches

We compared the predictability of our DrugSim2DR approach with other 3 state-of-the-art computational approaches for drug repurposing: CMap proposed by Lamb et al. [6], DvD, and SubtypeDrug. The CMap identified candidate drugs based on the reverse correlation between drug-induced gene expression and disease signatures. DvD provides a pipeline for drug repurposing by comparing gene signatures for drugs and diseases. SubtypeDrug was designed to perform the reverse correlation at the subpathway level. All 3 approaches used drugs from the CMap database as the background set for repurposing. However, no true-positive drugs were found for RCC and HNS in this background. Thus, we only compared DrugSim2DR with the other 3 approaches in the BRC, LUA, and TPLL datasets, respectively. According to the ROC curve analysis, DrugSim2DR showed better AUROC values than the other approaches in these datasets (Fig. 4B). In addition to AUROC, it is crucial that candidate drugs have sufficient research support for their disease-inhibiting capabilities. Therefore, we compared the number of evidence-supported drugs among the top *n* drugs identified by different approaches and DrugSim2DR, where *n* is equal to the number of candidate drugs identified by DrugSim2DR. As shown in Supplementary Tables S2–S4, DrugSim2DR can identify the highest number of evidence-supported candidate drugs for different diseases. This further underscores the excellent predictive capability of our approach.

#### Robustness analysis of DrugSim2DR

**Figure 5:**
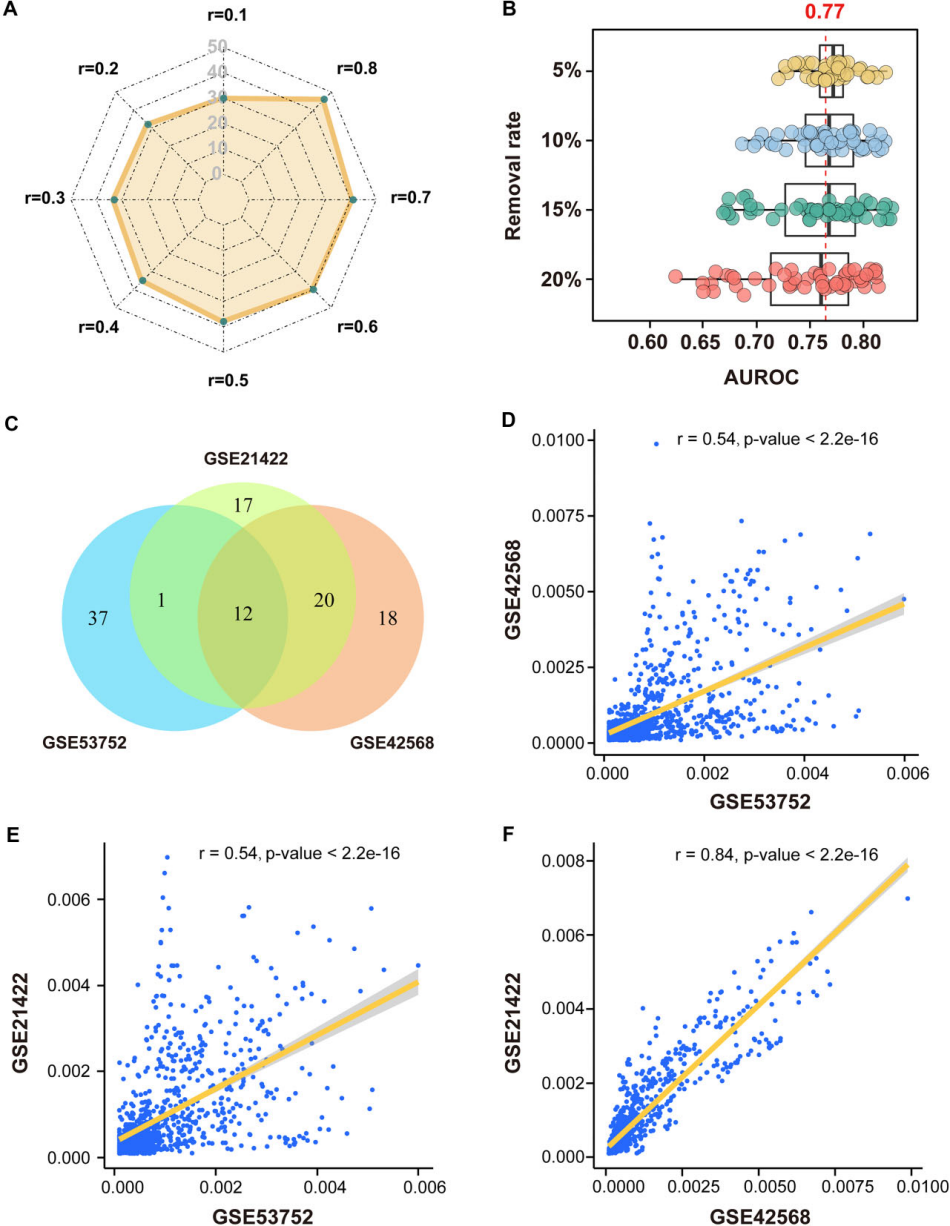
Robustness and reproducibility analysis of DrugSim2DR on breast cancer dataset. (A) Radar chart showing the overlapped number of top 50 drugs identified based on the restart probability *r* values set from 0.1 to 0.8 compared with that of *r* = 0.9. (B) Boxplots showing the AUROC values of predicted drugs for the different data removal. The red line indicates the AUROC value of the original data. (C) Venn diagram showing the overlapped number of the top 50 drugs identified in the GSE53752, GSE42568, and GSE21422 datasets. Correlation analysis of DrugSim2DR’s predictions for breast cancer across different datasets: (D) GSE53752 and GSE42568, (E) GSE53752 and GSE21422, and (F) GSE42568 and GSE21422.

In this study, we utilized the random walk with restart algorithm to calculate the eigenvector centrality for drug repurposing. In this algorithm, *r* represents the restart probability, meaning the probability that a node returns to the source node during the random walk, and we set *r* = 0.9. To confirm the influence of restart probability on the results, we respectively applied DrugSim2DR with the *r* values set from 0.1 to 0.8 at 0.1 intervals to the BRC and the LUA and then compared the top 50 drugs identified based on these *r* values with that of *r* = 0.9. The result for the BRC dataset showed that the percentages of overlapped drugs increased with *r* slowly and all above 60% (30/50) for the different *r* values (Fig. 5A), proving the robustness of DrugSim2DR’s results to changes in the restart probability. For the LUA dataset, we obtained similar results (Supplementary Fig. S3A).

To further assess the robustness of the DrugSim2DR, we performed the data removal tests using the BRC and LUA datasets. We removed the gene expression values from 5% to 20% at 5% intervals and reapplied the DrugSim2DR approach 50 times for each removal. For each removal, we performed the ROC analysis and calculated the AUROC values. The results showed that the different data removals had only a weak effect on the accuracy of the predictions. The median AUROC value remained above 0.75, even after the removal of up to 20% of the gene expression data in the BRC and LUA datasets (Fig. 5B and Supplementary Fig. S3B), indicating that the DrugSim2DR approach is robust to data removal.

#### Reproducibility analysis of DrugSim2DR

Due to the presence of intratumor heterogeneity and the impact of different platforms on sequencing results, the reproducibility of the approach’s results in different datasets of the same disease is critical. To evaluate the reproducibility of DrugSim2DR, we downloaded another 2 BRC gene expression datasets from the GEO database: GSE42568 and GSE21422. We applied DrugSim2DR to these datasets and compared their results with that of the initial GSE53752 dataset. To provide a general comparison, the top 50 drugs for each ranked drug list were used. As shown in Fig. 5C, 12 overlapped drugs were found among the 3 datasets. To further demonstrate the reproducibility of DrugSim2DR, we chose to perform a comprehensive consistency validation of the results. Specifically, we conducted correlation analysis on the 3 entire drug centrality score lists. The outcomes unveiled pairwise statistically significant high correlations (Fig. 5D–F, Pearson correlation test *P* < 2.2e-16). In the same way, we also performed the reproducibility validation on 3 LUA datasets: GSE68465, GSE74706, and GSE31210. Supplementary Fig. S3C exhibited that the overlapping ratios of drugs in these 3 lung cancers are 46% (23/50). The drug centrality scores optimized by DrugSim2DR in different lung cancer datasets still exhibit a strong correlation (Supplementary Fig. S3D–F, Pearson correlation test *P* < 2.2e-16). These results demonstrate the reproducibility of our approach for the different datasets of the same disease.

## Discussion

With the increasing availability of high-throughput sequencing technologies and bioinformatics, computational approaches to drug repurposing are gaining popularity compared to traditional experimental methods [41, 42]. In this study, we propose a network-based drug repurposing approach, DrugSim2DR, to identify cancer candidate drugs. Considering that different cancers have different molecular signatures, our DrugSim2DR approach innovatively constructs a drug–drug functional similarity network, where the edges are augmented with measurements of transcriptional dysregulation specific to a disease of interest. In DrugSim2DR, we first evaluated the functional similarity between drugs based on their shared functional gene sets (GO terms) while considering disease-specific transcriptional dysregulation levels of genes. Thus, 2 drugs with different physicochemical properties may be also identified as functionally similar in the specific disease environment, which enables more accurate prediction of drug–drug relationships. Our drug–drug functional similarity assessment may provide a new reference for prediction of drug combinations. We then constructed a weighted drug–drug functional similarity network, where the weighted edges reflect the disease-induced transcriptional dysregulation of all GO terms shared between the drugs. Drugs are more central in the network the more likely they may potentially treat the disease. The random walk with restart algorithm was used to calculate the centrality scores of drug nodes to evaluate how central each drug is in the network. Those drugs with significant centralities are reported as having potential therapeutic effects on the disease in their latent influence on the cascade of transcriptionally altered.

To evaluate the performance of our approach, we first calculated the drug–drug functional similarity scores in the context of breast cancer. Subsequently, we compared our predictions with those predicted by the other classical measures, which have chemical structure- and semantic-based similarity. The results indicate that our predicted drug–drug functional similarity is reliable and biologically meaningful (Fig. 2A, B). Despite the overall consistency with other measures, our approach identified 3 drug pairs with highly functional similarities in the context of breast cancer that are underestimated by other measures (Supplementary Fig. S1). And we observed these drug pairs were associated with molecular functions associated with breast cancer, such as hormone- and MHC (Major Histocompatibility Complex)-related function terms (Fig. 2C–E). This insightful prediction in the context of disease is the advantage of our approach over common similarity assessment measures. We next used the functional similarity scores to construct a drug–drug functional similarity network and used the random walk algorithm with restart to calculate the centrality scores of drugs. The drugs with significant centralities are reported as potentially anti–breast cancer drugs. To further demonstrate the predictive power of our approach, we applied it to datasets from multiple cancer types and performed ROC curve analysis. We also compared the predictive power of our approach with other drug repurposing approaches in multiple datasets. The results suggest that our approach could achieve excellent predictive performance in different cancers (Fig. 4A, B). Finally, the results of the reproducibility and robustness analysis confirm the reliability and credibility of DrugSim2DR and provide a solid foundation for its application in future research (Fig. 5 and Supplementary Fig. S3).

In this study, we developed a novel computational approach named DrugSim2DR to identify cancer candidate drugs based on a weighted drug–drug functional similarity network in the context of a specific disease. This approach holds the potential to augment and enhance the current landscape of computational drug repurposing strategies, thereby making valuable contributions to the field of drug discovery. To enable researchers to use our approach, we created an R-based software package, DrugSim2DR, which is freely available for download on CRAN [43].

## Availability of Source Code and Requirements

Project name: DrugSim2DR

Project homepage: https://CRAN.R-project.org/package=DrugSim2DR or https://github.com/hanjunwei-lab/DrugSim2DR

Operating system(s): Platform independent

Programming language: R 3.6 or higher

Other requirements: R packages igraph, stats, pheatmap, ChemmineR, rvest, base, sp, tidyr, reshape2, fastmatch.

License: GPL 2.0 or higher

BioTools ID: biotools:drugsim2dr_0.1.1

RRID: SCR_024564

## Abbreviations

AUROC: area under the ROC curve; BRC: breast cancer;CMap: Connectivity Map; DE: differential expression; DvD: Drug versus Disease; EMT: epithelial–mesenchymal transition; FDR: false discovery rate; GEO: Gene Expression Omnibus; GO: Gene Ontology; HNS: head and neck squamous carcinoma; LUA: lung cancer; MF: molecular function; RCC: renal cell carcinoma; ROC: receiver operating characteristic; TPLL: T-cell prolymphocytic leukemia.

## Supplementary Material

giad104_GIGA-D-23-00219_Original_Submission

giad104_GIGA-D-23-00219_Revision_1

giad104_Response_to_Reviewer_Comments_Original_Submission

giad104_Reviewer_1_Report_Original_Submission Xiaochen Bo -- 9/26/2023 Reviewed

giad104_Reviewer_2_Report_Original_Submission Dongqing Wei -- 10/6/2023 Reviewed

giad104_Reviewer_2_Report_Revision_1 Dongqing Wei -- 10/24/2023 Reviewed

giad104_Supplemental_Files

## Data Availability

Transcriptomic data for various diseases were retrieved from GEO [17]. The datasets supporting the results of this article, including drug–target interaction information, molecular functional gene sets, and core code, are available in the DrugSim2DR package from CRAN [43]. All supporting data and materials are available in the *GigaScience* GigaDB database [44].

## Additional Files

**Supplementary Text 1**. Details for calculating chemical structure-based and semantic-based drug–drug similarity.

**Supplementary Fig. S1**. Kernel density curve of chemical structure- and semantic-based drug–drug similarity scores.

**Supplementary Fig. S2**. Heatmap of gene expression levels of drugs’ targets between lung cancer and normal samples.

**Supplementary Fig. S3**. Robustness and reproducibility analysis of DrugSim2DR on lung cancer dataset.

**Supplementary Table S1**. Candidate drugs for lung cancer identified by DrugSim2DR with FDR <0.1.

**Supplementary Tables S2–S4**. Comparison of candidate drugs identified by different approaches.
