# Supplementary material for: DrugSim2DR: systematic prediction of drug functional similarities in the context of specific disease for drug repurposing
Source: Gigascience. 2023 Dec 19;12:giad104. doi: 10.1093/gigascience/giad104 (PMC10729734; doi:10.1093/gigascience/giad104)
Supplement: giad104_GIGA-D-23-00219_Original_Submission [file giad104_giga-d-23-00219_original_submission.pdf]

GigaScience

DrugSim2DR: Systematic prediction of drug functional similarities in the context of specific disease for drug repurposing

--Manuscript Draft--

**Manuscript Number:**	GIGA-D-23-00219
**Full Title:**	DrugSim2DR: Systematic prediction of drug functional similarities in the context of specific disease for drug repurposing
**Article Type:**	Research
**Funding Information:**	National Natural Science Foundation of China (62072145) — Dr. Junwei Han<br>Natural Science Foundation of Heilongjiang Province (LH2019C042) — Dr. Junwei Han
**Abstract:**	Traditional approaches to drug development are costly and involve high risks. The drug repurposing approach can be a valuable alternative to traditional approaches and has therefore received considerable attention in recent years. Herein, we develop a previously undescribed computational approach, called DrugSim2DR, which uses a network diffusion algorithm to identify candidate anti-cancer drugs based on a drug functional similarity network. The innovation of the approach lies in the drug-drug functional similarity network constructed in a manner that implicitly links drugs through their common biological functions in the context of a specific disease state, as the similarity relationships based on general states (e.g., network proximity or Jaccard index of drug targets) ignore disease-specific molecular characteristics. The drug functional similarity network may provide a reference for prediction of drug combinations. We describe and validate the DrugSim2DR approach through analysis of data on breast cancer and lung cancer. DrugSim2DR identified some FDA-approved anti-cancer drugs, as well as some candidate drugs validated by previous studies in the literature. Moreover, DrugSim2DR showed excellent predictive performance, as evidenced by ROC analysis and multi-approach comparisons in various cancer datasets. To increase the usability of our approach, we have developed an R-based software package, DrugSim2DR, which is freely available on CRAN (https://CRAN.R-project.org/package=DrugSim2DR).
**Corresponding Author:**	Junwei Han<br>Harbin Medical University<br>Harbin, China CHINA
**Corresponding Author Secondary Information:**	
**Corresponding Author's Institution:**	Harbin Medical University
**Corresponding Author's Secondary Institution:**	
**First Author:**	Jiashuo Wu
**First Author Secondary Information:**	
**Order of Authors:**	Jiashuo Wu
	Ji Li
	Yalan He
	Xilong Zhao
	Junling Huang
	Bingyue Pan
	Liang Cheng
	Junwei Han

Powered by Editorial Manager® and ProduXion Manager® from Aries Systems Corporation

Order of Authors Secondary Information:	
Additional Information:	
Question	Response
Are you submitting this manuscript to a special series or article collection?	No
**Experimental design and statistics**<br><br>Full details of the experimental design and statistical methods used should be given in the Methods section, as detailed in our Minimum Standards Reporting Checklist. Information essential to interpreting the data presented should be made available in the figure legends.<br><br>Have you included all the information requested in your manuscript?	Yes
**Resources**<br><br>A description of all resources used, including antibodies, cell lines, animals and software tools, with enough information to allow them to be uniquely identified, should be included in the Methods section. Authors are strongly encouraged to cite Research Resource Identifiers (RRIDs) for antibodies, model organisms and tools, where possible.<br><br>Have you included the information requested as detailed in our Minimum Standards Reporting Checklist?	Yes
**Availability of data and materials**<br><br>All datasets and code on which the conclusions of the paper rely must be either included in your submission or deposited in publicly available repositories (where available and ethically appropriate), referencing such data using a unique identifier in the references and in the "Availability of Data and Materials" section of your manuscript.	Yes

Have you have met the above requirement as detailed in our Minimum Standards Reporting Checklist?	

Powered by Editorial Manager® and ProduXion Manager® from Aries Systems Corporation

Manuscript Click here to access/download;Manuscript;manuscript.docx

DrugSim2DR: Systematic prediction of drug functional similarities in the context of specific disease for drug repurposing

Jiashuo Wu[1,†], Ji Li[1,†], Yalan He[1,†], Xilong Zhao[1], Junling Huang[1], Bingyue Pan[1], Liang Cheng[1,*], Junwei Han[1,*]

[1]College of Bioinformatics Science and Technology, Harbin Medical University, Harbin 150081, China.

[†]The authors should be regarded as joint First Authors.

* Corresponding Author: Junwei Han, College of Bioinformatics Science and Technology, Harbin Medical University, Harbin 150081, China, E-mail: hanjunwei@ems.hrbmu.edu.cn; Liang Cheng, College of Bioinformatics Science and Technology, Harbin Medical University, Harbin 150081, China, E-mail: liangcheng@hrbmu.edu.cn;

Abstract

Traditional approaches to drug development are costly and involve high risks. The drug repurposing approach can be a valuable alternative to traditional approaches and has therefore received considerable attention in recent years. Herein, we develop a previously undescribed computational approach, called DrugSim2DR, which uses a network diffusion algorithm to identify candidate anti-cancer drugs based on a drug functional similarity network. The innovation of the approach lies in the drug-drug functional similarity network constructed in a manner that implicitly links drugs through their common biological functions in the context of a specific disease state, as the similarity relationships based on general states (e.g., network proximity or Jaccard index of drug targets) ignore disease-specific molecular characteristics. The drug functional similarity network may provide a reference for prediction of drug combinations. We describe and validate the DrugSim2DR approach through analysis of data on breast cancer and lung cancer. DrugSim2DR identified some FDA-approved anti-cancer drugs, as well as some candidate drugs validated by previous studies in the literature. Moreover, DrugSim2DR showed excellent predictive performance, as evidenced by ROC analysis and multi-approach comparisons in various cancer datasets. To increase the usability of our approach, we have developed an R-based software package, DrugSim2DR, which is freely available on CRAN (https://CRAN.R-project.org/package=DrugSim2DR).

Key words: computational drug repurposing; drug-drug similarity; network analysis; specific disease state

Background

Cancer is one of the most common complex diseases in terms of morbidity and

mortality, Because of the complexity and diversity of cancer, finding effective therapeutic drugs for cancer patients remains a formidable challenge. At present, there is widespread interest among researchers in drug repurposing methods. Drug repurposing is defined as the process of applying an existing drug ingredient to a new indication [1]. Compared to traditional drug discovery, drug repurposing has higher efficiency and lower risk due to a more comprehensive understanding of the safety and toxicity profiles of existing drugs. In addition, since the drug has already gone through clinical trials, it has a higher probability of being approved for use in the clinic [2]. In recent years, researchers have focused on drug repurposing in silico approaches [3]. With advances in sequencing technology and the availability of large amounts of molecular omics data, there is a better prospect of systematically inferring the new relationship between drugs and/or diseases [1], which will facilitate the development of computational drug repurposing approaches.

Several computational techniques have been documented for drug repurposing, drawing upon expertise in bioinformatics and systems biology. For example, Connectivity Map (CMap) is a groundbreaking algorithm proposed by Lamb et al. in 2006 [4]. Through in-depth analysis of reverse association between drug-induced and disease-induced gene expression profiles, CMap unveils valuable insights into the underlying mechanisms and offers new avenues for drug repurposing. Subsequently, Drug versus Disease (DvD) [5] improves the CMap approach and performs drug repurposing by comparing drug and disease gene-based signatures and evaluating their reverse relationship. SubtypeDrug identifies abnormal subpathways induced by diseases and drugs respectively, and then evaluates the reverse correlation between drugs and diseases at the subpathway level for drug repurposing [6]. DRviaSPCN repurposes drugs of cancer by considering drug-induced subpathways and their

crosstalk effect [3]. Such approaches generally perform drug repurposing by evaluating drug-disease reverse association at the gene expression or pathway activity level. Although these approaches effectively discover some cancer candidate drugs, they do not incorporate the similarities/interactions between drugs, which is a research area that has to be thoroughly explored [7]. PriorCD is a network-based drug repurposing approach that constructs a drug functional similarity network at the pathway level and uses a global network propagation algorithm to prioritize candidate cancer drugs [8]. Groza, V. et al. constructed a drug similarity network based on the drug-target interactions for drug repurposing [9]. PIMD predicts novel therapeutic uses of drugs based on a drug similarity network constructed by integrating chemical, pharmacological, and clinical data of drugs [10]. These network-based approaches achieved good results for drug repurposing; however, they generally constructed drug-drug similarity/interaction networks based on drug targets, chemical structures, and semantic similarity, etc., and neglected disease-specific molecular characteristics. Indeed, complex diseases, especially cancer, are highly heterogeneous, and different cancers exhibit varying molecular characteristics and biological processes during their development [11]. In different diseases, the drug similarities/interactions will be diverse. Therefore, it is indispensable to incorporate molecular characteristics in the context of a specific disease state for inferring drug-drug relationships and drug repurposing.

In this study, we presented a novel network-based approach, named DrugSim2DR, for drug repurposing based on a weighted drug-drug functional similarity network constructed with the transcriptionally altered genes in the context of the specific disease state. In the approach, we first constructed the drug-drug similarity network with the gene expression profiles between a pair of binary

conditions (e.g., case/control, normal/diseased). In the drug-drug similarity network, the links and their weights reflect both the extent to which the targets of drugs shared biological function and the differential transcriptional level of target genes common to those drugs. Drugs were prioritized according to the network centrality scores calculated by a network propagation algorithm, and then a bootstrap-based approach is used to estimate the statistical significance of the drug centrality scores. We applied DrugSim2DR to datasets of breast cancer and lung cancer and achieved better predictive performance that surpassed some other classical drug repurposing approaches, demonstrating the potential of our approach as a practical drug repurposing tool. We also developed a tool package called "DrugSim2DR" for implementing our approach, which could be freely available from https://CRAN.R-project.org/package=DrugSim2DR.

Materials and Methods

Data acquisition and processing

To construct a drug similarity network and perform the drug repurposing, we totally collected 5804 drugs with corresponding targets from the DrugBank database (https://go.drugbank.com/). We selectively retained drugs possessing a target gene count of over 3 but fewer than 500 and obtained 1289 drugs. Then, Molecular Function (MF) gene sets derived from Gene Ontology (GO) were downloaded from the Molecular Signature Database (https://www.gsea-msigdb.org/gsea/msigdb/index.jsp, version 7.5.1). To comprehensively assess and comprehend our methodology, we applied our approach to multiple types of cancer datasets. The transcriptional data of different cancer types were downloaded from the GEO database (https://www.ncbi.nlm.nih.gov/geo/), including breast cancer

(GSE53752[12], GSE42568[13], and GSE21422[14]), lung adenocarcinoma (GSE68465[15], GSE31210[16], and GSE74706[17]), T-cell prolymphocytic leukemia (GSE5788[18]), renal cell carcinoma (GSE53757[19]) and head and neck squamous carcinoma (GSE6631[20]). In these datasets, the expression values for each gene were standardized to a normal distribution using the z-score normalization method across all samples.

Calculating transcriptional dysregulation levels of genes

We estimated the transcriptional dysregulation level of genes in the context of a specific disease (Step 1 in Figure 1). For each gene, the t-test was applied to assess the gene differential expression extent between normal and disease samples. Then, we transformed the p-value of t-test of each gene to z-score through $z = \varphi^{-1}(1-p)$, where φ^{-1} is the inverse normal cumulative density function. We defined the absolute value of the z-score as the differential expression score (DE-score) to reflect the extent to which gene expression is affected by the disease. The higher the DE-score, the stronger the gene is transcriptionally dysregulated.

Constructing a *Drug/GO* bipartite network under the disease context

In step 2, we intended to build a bipartite network using drugs and GO gene sets (terms) as nodes (Step 2 in Figure 1). If the targets of a drug exist in a GO term, we believe that the GO term is related to this drug. We quantified the relationship between a drug (D) and a GO term (G) by a weighted undirected edge. The weight (W_{DG}) was defined as follows:

$$W_{DG} = J_{DG} \times med\{DE_x | x \in D \cap G\} \qquad (1)$$

$$J_{DG} = \frac{|D \cap G|}{|D \cup G|} \qquad (2)$$

J_{DG} is the Jaccard index, which was used to measure the degree of overlap between a GO term and a target gene set for a drug. We believe that the relationship between drugs and GO terms is not only decided by the overlap, but also by the transcriptional dysregulation of the overlapped genes in the disease context. Thus we calculated $med\{DE_x \mid x \in D \cap G\}$, the median DE-score of all genes common to targets of a drug and a GO term, which reflects the degree of transcriptional dysregulation in the disease state of overlapped genes. Finally, with W_{DG} as the weight of the edge between D and G, reflecting the disease-modulated D/G relationship, a D/G bipartite network was constructed. The final network is represented by an adjacency matrix W, in which rows are drugs, columns are GO terms, and elements represent the degree of association between drugs and GO terms.

Constructing a drug-drug functional similarity network

Calculating drug-drug functional similarity scores. In step 3, we first used the information from the D/G bipartite network to calculate the functional similarity score between two drugs (Step 3 in Figure 1). We consider that the functional similarity score between two drugs will be stronger when they shared more neighbor GO terms. Thus, we define the similarity score between a pair of drugs (D_a and D_b) as follows:

$$S_{ab} = \sum_{j=1}^{N_G} W_{D_a G_j} \times W_{D_b G_j} \qquad (3)$$

where N_G is the total number of shared GO terms between the two drugs; W_{DaGj} or W_{DbGj} is the edge weight between D_a or D_b and GO term G_j. That means the functional similarity score between D_a and D_b is the sum of the contributions, concerning the disease induced transcriptional dysregulation of all GO terms shared between them.

Constructing a drug-drug functional similarity network based on the D/G bipartite network. Furthermore, we constructed a drug-drug functional similarity network, in which the edge weights represent the similarity scores between drugs. To represent the drug-drug network, we defined an adjacency matrix *A*:

$$A = W \cdot W^T \qquad (5)$$

where *W* is the adjacency matrix of the *D/G* bipartite network. Thus, the elements of *A* correspond to the functional similarity scores (see formula 3) between drugs in the drug-drug similarity network. We assign the value of 0 to the diagonal element of matrix *A*, indicating self-links were removed. After these steps, a drug functional similarity network under a specific disease context is then constructed, which comprises a total of 1,289 drug nodes and 322,250 edges. This process was previously used to construct a cell-cell crosstalk network [21].

Prioritizing disease candidate drugs through a network propagation algorithm

In the drug-drug functional similarity network, the edge weight reflects both the drug functional similarity and the disease-induced transcriptional dysregulation. It is reasonable to assume that the evidence of a drug may potentially treat a disease could be reinforced by the evidence of its neighbors. Thus, a drug is more inclined to act on a disease if it is linked to more other neighbor nodes and the edges have larger weight. In step 4, we applied a network propagation algorithm, the random walk with restart, to calculate the eigenvector centrality score of drugs, which is a measure to determine the significance of disease candidate drugs. In this algorithm, the more central a drug node is, the more probable it is to be visited by the random walker and result in a larger eigenvector centrality score. To implement this algorithm, we defined a probability transition matrix *T* by row-normalizing the adjacency matrix *A*. The

formula is as follows:

$$T_{ab} = \frac{A_{ab}}{\sum_{a=1}^{N_D} A_{ab}} \qquad (6)$$

where N_D is the total number of drugs in the network; T_{ab} denotes the probability of transferring from D_a to D_b. The formula of random walk with restart algorithm is as follows:

$$v^{t+1} = (1-r)Tv^t + rv^0 \qquad (7)$$

where v^0 is the initial probability vector in which equal probabilities are assigned to all the nodes and the probability of all nodes is sum to 1; v^t is the probability vector comprising the probabilities of the nodes at step t; T is the probability transition matrix; r is the restart probability. It has been demonstrated to have only a slight effect on the results when it fluctuated between 0.1 and 0.9 [22], we set r=0.9 in the study. After a limited number of iterations, the probability vector v^t will converge to a stable state v (the difference between v^t and v^{t+1} is less than 10^{-10}). Its ith element v_i in the vector represents the eigenvector centrality score of drug D_i. A larger eigenvector centrality score of a drug suggests a potential therapeutic effect on the disease.

To evaluate the statistical significance (p-value) of drug centrality score, we performed the permutation test. The drug centrality score was calculated in the drug similarity network, which was constructed based on the intersection of each pair of drug target gene set and GO term and their transcriptional dysregulation scores, DE-scores. As the intersection is constant, and the DE-scores of genes are variable, we used bootstrap resampling of our original DE-scores at the gene level, and repeated step 2-4 to find statistical significance candidate drugs. This produced a vector of random drug centrality scores, v^*. By repeating this progress 1,000 times, a set of

randomly generated drug centrality score vectors were produced$\{v^{*1}, \cdots, v^{*1000}\}$. For given drug *D*$_i$, we compared its original drug centrality score v_i with the set of random scores $\{v_i^{*k}\}_{k=1}^{1000}$. And the *p-value* of drug *D*$_i$ was calculated as follows:

$$p - value\ (D_i) = \frac{\sum_{k=1}^{1000} I\ \{v_i{}^{*k} \geq v_i\}}{1000} \qquad (8)$$

where *I* is the indicator function. To correct multiple comparisons, the p-values were then adjusted using Benjamin and Hochberg's false discovery rate (FDR) method. We have developed an available CRAN package called "DrugSim2DR" for implementing our approach.

Results

Case study: Breast cancer

Calculating the drug-drug functional similarity scores in breast cancer. The study of drug-drug similarity has been used repeatedly in drug repurposing approaches. The current drug-drug similarity prediction approaches mainly depend on chemical structure- and semantic-based similarity. However, these approaches did not adopt the molecular features of disease, and the drug-drug similarity may be varied in different diseases. Our approach innovatively considers transcriptional dysregulation in the context of a specific disease. Using drug target gene sets, GO terms (Molecular Function), and the breast cancer gene expression profiles (GSE53752), we calculated the functional similarity score between each pair of drugs reflecting both the extent to which incident drug action in ways that produce similar biological outcomes and the differential transcriptional activity of genes common to those drugs (See Materials and Methods). To test if our drug-drug functional similarity was associated with the traditional chemical structure- or semantic-based similarity (Supplementary Text 1),

we categorized all drug pairs into four groups (Q1-Q4) according to the quartiles of our functional similarity scores. We found that our drug-drug functional similarities were significantly correlated with chemical structure similarities (Spearman's rank correlation test, $p<2.2e-16$) and semantic similarities (Spearman's rank correlation test, $p<2.2e-16$) respectively, and the drug pairs in the high functional similarity group have larger chemical structure similarities and semantic similarities compared with that of low functional similarity group (Figure 2A and B). The results demonstrated that our drug-drug functional similarity algorithm could identify biologically significant drug pairs.

To explain our results in detail, we highlighted the top 10 drug pairs with the highest similarity scores (Table 1). Notably, we discovered that some of these drug pairs also have larger chemical structure- and semantic-based similarities, such as fomepizole/pyrazole, benzthiazide/ellagic acid, and benzthiazide/cyclothiazide (Supplementary Figure S1A and B). These findings are in line with our expectations. Moreover, as our drug-drug functional similarity scores were calculated in the transcriptional profiling of breast cancer, we further tested if the scores could provide something new insight into drug-drug similarity. Interestingly, we observed that three drug pairs (ouabain/tegoprazan, ouabain/bisacodyl, and bisacodyl/tegoprazan) had comparatively lower chemical structure and semantic similarities (Supplementary Figure S1A and B) but showed large functional similarities in our algorithm (Table 1). This may be because they shared the same functional terms in the context of the breast cancer dataset. For example, ouabain and tegoprazan shared the same functional terms including “Hormone binding” and “Steroid-hormone binding” (Figure 2C). Moreover, most of these functional terms are associated with the development of breast cancer, which may result in their functional similarity.

Specifically, several studies have shown that some estrogens or progestins can exacerbate the proliferation of breast cancer cells, and targeting hormone receptor is a widely used and effective treatment strategy [23, 24]. Furthermore, the drug pairs for ouabain/bisacodyl and bisacodyl/tegoprazan showed similar results (Supplementary Figure S1A and B and Figure 2D and E). This indicates that our approach may identify something new functional similar drug pairs in the context of a specific disease, which may provide a new reference for prediction of drug combinations.

Drug repurposing for breast cancer. Furthermore, leveraging the functional similarity scores of all drug pairs in the context of breast cancer, we constructed a weighted drug-drug similarity network. Within this network, drug nodes are interconnected by edges with weights that directly correspond to the level of their functional similarity. A drug(s) may potentially treat the disease can be expected to be reinforced by the evidence of its neighbors and their linked weights. The random walk algorithm with restart was then applied to the network to calculate the eigenvector centrality scores of drugs. Drug nodes are more central the more likely they are to be visited in the random walk and the larger centrality scores they obtain. The significance of these centrality scores of drugs was assessed using a permutation test. With the default FDR<0.1, our DrugSim2DR approach identified 5 potential anti-breast cancer drugs (Table 2). An interesting observation is that fluoxymesterone, the top-ranked drug, has received FDA approval for breast cancer treatment. Fluoxymesterone could competitively block estrogen receptors, preventing the development of hormone-dependent tumor lines [25, 26]. Moreover, we also identified some potential candidate drugs with evidence that may inhibit the development of breast cancer. For example, gestrinone has been demonstrated to have anti-cancer effects in cell experiments, particularly in the field of gynecological cancer [23]. Pyrazole is a heterocyclic

organic compound and the effect of its derivative on breast cancer has been confirmed by multiple studies. Ashourpour et al. provided evidence that pyrazole derivatives can cause apoptosis in the MDA-MB-468 cells (breast cancer cell line) through the Reactive oxygen species [27]. Gutierrez et al. proved that a pyrazole-based derivative P3C has demonstrated resistance to breast cancer, meaning a great anticancer therapy [28]. Additionally, medroxyprogesterone acetate acts as a progestin-receptor agonist and suppresses the proliferation of cancer cells in ER+ breast cancer [29]. Furthermore, we tested the expression levels of target genes of the above drugs between breast cancer and normal samples, and they exhibited contrasting expression differences (Figure 3). This suggests that these drugs may intervene in the disease process by regulating target expression and may become meaningful treatment options.

Case study: lung cancer

We then used a lung cancer dataset (GSE68465) to illustrate the effectiveness of DrugSim2DR in prioritizing cancer candidate drugs. With FDR<0.1, DrugSim2DR identified 9 potential anti-lung cancer drugs (Supplementary Table S1). Interestingly, we identified two FDA-approved drugs for the treatment of lung cancer, which are methotrexate and pemetrexed. Moreover, some candidate drugs with positive evidence have also been identified. For example, seliciclib is a potent CDK inhibitor that is currently undergoing phase-2 clinical testing in lung and B-cell malignancies. It has shown the ability of seliciclib to induce cell-cycle arrest and apoptosis in cancer cells [30]. Olomoucine is a cyclin-dependent kinase inhibitor. Some available studies have confirmed that olomoucine can inhibit the G1/S transition of cells and thus inhibit the proliferation of cancer cells [31, 32]. SU9516 is a cyclin-dependent kinase 2 (CDK2) inhibitor, exhibiting an inhibitory effect on Epithelial-mesenchymal

transition (EMT) in A549 lung cancer cells. Specifically in tumor cell emergence and migration, EMT is a significant pathogenic process in cancer [33]. This ability to inhibit EMT predicts the potential of SU9516 as a lung cancer drug candidate. Alvocidib effectively inhibits EML4-ALK cells, driving lung cancer progression, thereby suppressing tumor growth, and inducing apoptosis [34]. Alvocidib and docetaxel are in phase II clinical trials for the treatment of non-small cells [35]. We also tested the expression levels of target genes of the evidence-supported drugs between lung cancer and normal samples, and they exhibited contrasting expression differences (Supplementary Figure S2).

These results indicate that DrugSim2DR can offer precise and varied treatment choices to patients. It can expedite drug discovery and furnish novel prospects and opportunities for subsequent research and clinical application.

Evaluating the DrugSim2DR approach

Performance of the DrugSim2DR approach. To evaluate the reliability of the DrugSim2DR approach, we performed the Receiver Operating Characteristic (ROC) curve analysis according to the centrality scores of drugs. The FDA-approved drugs for the specific cancer types were collected from the DrugBank database (https://www.drugbank.ca/), and were used as the true-positive drug set. In the above breast cancer (BRC) and lung cancer (LUA) datasets, the values of area under the ROC curve (AUROC) reached 0.77 and 0.76 respectively (Figure 4A). To confirm the accuracy and wide applicability of DrugSim2DR more comprehensively, we then applied it to three other cancer gene expression datasets, T-cell prolymphocytic leukemia (TPLL), renal cell carcinoma (RCC), and head and neck squamous carcinoma (HNS) (see Materials and Methods). As shown in Figure 4A, the values of AUROC of our approach applied to the TPLL was 0.73, to the RCC was 0.77, and to

the HNS was 0.86. These results indicate that DrugSim2DR could effectively identify cancer candidate drugs.

Comparison of DrugSim2DR with other approaches. We compared the predictability of our DrugSim2DR approach with other three state-of-the-art computational approaches for drug repurposing: CMap proposed by Lamb et al., DvD and SubtypeDrug. The CMap identified candidate drugs based on the reverse correlation between drug-induced gene expression and disease signatures. DvD provides a pipeline for drug repurposing by comparing gene signatures for drugs and diseases. SubtypeDrug was designed to perform the reverse correlation at the subpathway level. All three approaches used drugs from the CMap database as the background set for repurposing. However, no true-positive drugs were found for RCC and HNS in this background. Thus, we only compared DrugSim2DR with the other three approaches in the BRC, LUA, and TPLL datasets respectively. According to the ROC curve analysis, DrugSim2DR showed better AUROC values than the other approaches in these datasets (Figure 4B).

Robustness analysis of DrugSim2DR. In this study, we utilized the random walk with restart algorithm to calculate the eigenvector centrality for drug repurposing. In this algorithm, r represents the restart probability, meaning the probability that a node returns to the source node during the random walk, and we set r=0.9. To confirm the influence of restart probability on the results, we respectively applied DrugSim2DR with the r values set from 0.1 to 0.8 at 0.1 intervals to the BRC and the LUA, and then compared the top 50 drugs identified based on these r values with that of r=0.9. The result for the BRC dataset showed that the percentages of overlapped drugs increased with r slowly and all above 60% (30/50) for the different r values (Figure 5A), proving the slight effect of restart probability on the results. For the LUA dataset, we

obtained similar results (Supplementary Figure S3A).

Due to the presence of intra-tumor heterogeneity and the impact of different platforms on sequencing results, the reproducibility of the approach's results in different datasets of the same disease is critical. To evaluate the the replicability of DrugSim2DR, we downloaded another two BRC gene expression datasets from the GEO database: GSE42568 and GSE21422. We applied DrugSim2DR to these datasets and compared their results with that of the initial GSE53752 dataset. To provide a general comparison, the top 50 drugs for each ranked drug list were used. As shown in Figure 5B, 12 overlapped drugs were found among the three datasets. In the same way, we also performed the reproducibility validation on three LUA datasets: GSE68465, GSE74706 and GSE31210. The Supplementary Figure S3B exhibited that the overlapping ratios of drugs in these three lung cancers are 46% (23/50) drugs, which demonstrates the reproducibility of our method for the different datasets of the same cancer.

Furthermore, to assess the robustness of the DrugSim2DR approach, we performed the data removal tests using the BRC and LUA datasets. We removed the gene expression values from 5% to 20% at 5% intervals and reapplied the DrugSim2DR approach 20 times for each removal. For each removal, we performed the ROC analysis and calculated the AUROC values. The results showed that the different data removals had only a weak effect on the accuracy of the predictions. The median AUROC value remained above 0.7, even after the removal of up to 20% of the gene expression data in the BRC and LUA datasets (Figure 5C and Supplementary Figure S3C), indicating that the DrugSim2DR approach is robust to data removal.

Discussion

With the increasing availability of high-throughput sequencing technologies and bioinformatics, computational approaches to drug repurposing are gaining popularity compared to traditional experimental methods [36]. In this study, we propose a network-based drug repurposing approach, DrugSim2DR, to identify cancer candidate drugs. Considering that different cancers have different molecular signatures, our DrugSim2DR approach innovatively constructs a drug-drug functional similarity network, where the edges are augmented with measurements of transcriptional dysregulation specific to a cancer of interest. In DrugSim2DR, we first evaluated the functional similarity between drugs based on their shared functional gene sets (GO terms) while considering disease-specific transcriptional dysregulation levels of genes. Thus, two drugs with different physicochemical properties may be also identified as functionally similar in the specific disease environment, which enables more accurate prediction of drug-drug relationships. Our drug-drug functional similarity assessment may provide a new reference for prediction of drug combinations. We then constructed a weighted drug-drug functional similarity network, where the weighted edges reflect the disease-induced transcriptional dysregulation of all GO terms shared between the drugs. Drugs are more central in the network the more likely they may potentially treat the disease. The random walk with restart algorithm was used to calculate the centrality scores of drug nodes to evaluate how central each drug is in the network. Those drugs with significant centralities are reported as potential therapeutic effects on the disease in their latent influence on the cascade of transcriptionally altered.

To evaluate the performance of our approach, we first calculated the drug-drug functional similarity scores in the context of breast cancer. Subsequently, we compared our predictions with those predicted by the other classical measures, which

are chemical structure- and semantic-based similarity. The results indicate that our predicted drug-drug functional similarity is reliable and biologically meaningful (Figure 2A and B). Despite the overall consistency with other measures, our approach identified three drug pairs with highly functional similarities in the context of breast cancer that are underestimated by other measures (Supplementary Figure S1). And we observed these drug pairs were associated with molecular functions associated with breast cancer, such as hormone- and MHC-related function terms (Figure 2C-E). This insightful prediction in the context of disease is the advantage of our approach over common similarity assessment measures. We next used the functional similarity scores to construct a drug-drug functional similarity network and used random walk algorithm with restart to calculate the centrality scores of drugs. The drugs with significant centralities are reported as potentially anti-breast cancer drugs. To further demonstrate the predictive power of our approach, we applied it to datasets from multiple cancer types and performed ROC curve analysis. We also compared the predictive power of our approach with other drug repurposing approaches in multiple datasets. The results suggest that our approach could achieve excellent predictive performance in different cancers (Figure 4A and B). Finally, the results of the reproducibility and robustness analysis confirm the reliability and credibility of DrugSim2DR, and provide a solid foundation for its application in future research (Figure 5 and Supplementary Figure S3).

In this study, we developed a novel computational approach named DrugSim2DR to identify cancer candidate drugs based on a weighted drug-drug functional similarity network in the context of a specific disease. This approach holds the potential to augment and enhance the current landscape of computational drug repurposing strategies, thereby making valuable contributions to the field of drug

discovery. To enable researchers to use our approach, we created an R-based software package, DrugSim2DR, which is freely available for download on CRAN (https://CRAN.R-project.org/package=DrugSim2DR).

Availability of Source Code and Requirements

Project name: DrugSim2DR

Project homepage: https://CRAN.R-project.org/package=DrugSim2DR or https://github.com/hanjunwei-lab/DrugSim2DR

Operating system(s): Platform independent

Programming language: R 3.6 or higher

Other requirements: R packages igraph, stats, pheatmap, ChemmineR, rvest, base, sp, tidyr, reshape2, fastmatch.

License: GPL 2.0 or higher

Data Availability

Transcriptomic data for various diseases were retrieved from the Gene Expression Omnibus (GEO, https://www.ncbi.nlm.nih.gov/geo/). The data sets supporting the results of this article including drug-targets interaction information, molecular functional gene sets, and core code are available in the DrugSim2DR package from CRAN (https://CRAN.R-project.org/package=DrugSim2DR).

Additional Files

Supplementary Text 1. Details for calculating chemical structure-based and Semantic-based drug-drug similarity.

Supplementary Figure S1. Kernel density curve of chemical structure- and semantic-based drug-drug similarity scores.

Supplementary Figure S2. Heatmap of gene expression levels of drugs' targets between lung cancer and normal samples.

Supplementary Figure S3. Robustness analysis of DrugSim2DR on lung cancer dataset.

Supplementary Table S1. Candidate drugs for lung cancer identified by DrugSim2DR with FDR < 0.1.

Abbreviations

CMap: Connectivity Map; DvD: Drug versus Disease; MF: molecular function; GO: Gene Ontology; DE-score: differential expression score; GEO: Gene Expression Omnibus; FDR: false discovery rate; CDK2: cyclin-dependent kinase 2; EMT: Epithelial-mesenchymal transition; ROC: Receiver Operating Characteristic; AUROC: area under the ROC curve; BRC: breast cancer; LUA: lung cancer; TPLL: T-cell prolymphocytic leukemia; RCC: renal cell carcinoma; HNS: head and neck squamous carcinoma.

Competing Interests

The authors declare that they have no competing interests.

Funding

National Natural Science Foundation of China (grant no.62072145), the Natural Science Foundation of Heilongjiang Province (grant no. LH2019C042).

Authors' Contribution

Conceptualization, J.H. and J.W.; methodology, J.L. and J.W.; validation, J.L. and Y.H.; formal analysis, Y.H. and X.Z.; investigation, J.W. and J.H.; resources, J.H. and B.P.; data curation, X.Z.; writing—original draft preparation, J.L. and J.W.; writing—review and editing, J.H. and L.C.; visualization, B.P.; supervision, J.H.; project administration, J.H.; funding acquisition, J.H. All authors have read and agreed to the published version of the manuscript. Authorship must be limited to those who have contributed substantially to the work reported.

References

1. Sadegh S, Skelton J, Anastasi E, Bernett J, Blumenthal DB, Galindez G, et al. Network medicine for disease module identification and drug repurposing with the NeDRex platform. Nat Commun. 2021;12 1:6848. doi:10.1038/s41467-021-27138-2.
2. Jourdan JP, Bureau R, Rochais C and Dallemagne P. Drug repositioning: a brief overview. J Pharm Pharmacol. 2020;72 9:1145-51. doi:10.1111/jphp.13273.
3. Wu J, Li X, Wang Q and Han J. DRviaSPCN: a software package for drug repurposing in cancer via a subpathway crosstalk network. Bioinformatics. 2022;38 21:4975-7. doi:10.1093/bioinformatics/btac611.
4. Lamb J, Crawford ED, Peck D, Modell JW, Blat IC, Wrobel MJ, et al. The Connectivity Map: using gene-expression signatures to connect small molecules, genes, and disease. Science. 2006;313 5795:1929-35. doi:10.1126/science.1132939.
5. Pacini C, Iorio F, Goncalves E, Iskar M, Klabunde T, Bork P, et al. DvD: An R/Cytoscape pipeline for drug repurposing using public repositories of gene

expression data. Bioinformatics. 2013;29 1:132-4. doi:10.1093/bioinformatics/bts656.

6. Han X, Kong Q, Liu C, Cheng L and Han J. SubtypeDrug: a software package for prioritization of candidate cancer subtype-specific drugs. Bioinformatics. 2021;37 16:2491-3. doi:10.1093/bioinformatics/btab011.

7. Lotfi Shahreza M, Ghadiri N, Mousavi SR, Varshosaz J and Green JR. A review of network-based approaches to drug repositioning. Brief Bioinform. 2018;19 5:878-92. doi:10.1093/bib/bbx017.

8. Di J, Zheng B, Kong Q, Jiang Y, Liu S, Yang Y, et al. Prioritization of candidate cancer drugs based on a drug functional similarity network constructed by integrating pathway activities and drug activities. Mol Oncol. 2019;13 10:2259-77. doi:10.1002/1878-0261.12564.

9. Groza V, Udrescu M, Bozdog A and Udrescu L. Drug Repurposing Using Modularity Clustering in Drug-Drug Similarity Networks Based on Drug-Gene Interactions. Pharmaceutics. 2021;13 12 doi:10.3390/pharmaceutics13122117.

10. He S, Wen Y, Yang X, Liu Z, Song X, Huang X, et al. PIMD: An Integrative Approach for Drug Repositioning Using Multiple Characterization Fusion. Genomics Proteomics Bioinformatics. 2020;18 5:565-81. doi:10.1016/j.gpb.2018.10.012.

11. Dagogo-Jack I and Shaw AT. Tumour heterogeneity and resistance to cancer therapies. Nat Rev Clin Oncol. 2018;15 2:81-94. doi:10.1038/nrclinonc.2017.166.

12. Kuo WH, Chang YY, Lai LC, Tsai MH, Hsiao CK, Chang KJ, et al. Molecular characteristics and metastasis predictor genes of triple-negative breast cancer: a clinical study of triple-negative breast carcinomas. PLoS One. 2012;7 9:e45831.

doi:10.1371/journal.pone.0045831.

13. Clarke C, Madden SF, Doolan P, Aherne ST, Joyce H, O'Driscoll L, et al. Correlating transcriptional networks to breast cancer survival: a large-scale coexpression analysis. Carcinogenesis. 2013;34 10:2300-8. doi:10.1093/carcin/bgt208.

14. Kretschmer C, Sterner-Kock A, Siedentopf F, Schoenegg W, Schlag PM and Kemmner W. Identification of early molecular markers for breast cancer. Mol Cancer. 2011;10 1:15. doi:10.1186/1476-4598-10-15.

15. Director's Challenge Consortium for the Molecular Classification of Lung A, Shedden K, Taylor JM, Enkemann SA, Tsao MS, Yeatman TJ, et al. Gene expression-based survival prediction in lung adenocarcinoma: a multi-site, blinded validation study. Nat Med. 2008;14 8:822-7. doi:10.1038/nm.1790.

16. Okayama H, Kohno T, Ishii Y, Shimada Y, Shiraishi K, Iwakawa R, et al. Identification of genes upregulated in ALK-positive and EGFR/KRAS/ALK-negative lung adenocarcinomas. Cancer Res. 2012;72 1:100-11. doi:10.1158/0008-5472.CAN-11-1403.

17. Marwitz S, Depner S, Dvornikov D, Merkle R, Szczygiel M, Muller-Decker K, et al. Downregulation of the TGFbeta Pseudoreceptor BAMBI in Non-Small Cell Lung Cancer Enhances TGFbeta Signaling and Invasion. Cancer Res. 2016;76 13:3785-801. doi:10.1158/0008-5472.CAN-15-1326.

18. Durig J, Bug S, Klein-Hitpass L, Boes T, Jons T, Martin-Subero JI, et al. Combined single nucleotide polymorphism-based genomic mapping and global gene expression profiling identifies novel chromosomal imbalances, mechanisms and candidate genes

important in the pathogenesis of T-cell prolymphocytic leukemia with inv(14)(q11q32). Leukemia. 2007;21 10:2153-63. doi:10.1038/sj.leu.2404877.

19. von Roemeling CA, Radisky DC, Marlow LA, Cooper SJ, Grebe SK, Anastasiadis PZ, et al. Neuronal pentraxin 2 supports clear cell renal cell carcinoma by activating the AMPA-selective glutamate receptor-4. Cancer Res. 2014;74 17:4796-810. doi:10.1158/0008-5472.CAN-14-0210.

20. Kuriakose MA, Chen WT, He ZM, Sikora AG, Zhang P, Zhang ZY, et al. Selection and validation of differentially expressed genes in head and neck cancer. Cell Mol Life Sci. 2004;61 11:1372-83. doi:10.1007/s00018-004-4069-0.

21. Sheng Y, Wu J, Li X, Qiu J, Li J, Ge Q, et al. iATMEcell: identification of abnormal tumor microenvironment cells to predict the clinical outcomes in cancer based on cell-cell crosstalk network. Brief Bioinform. 2023;24 2 doi:10.1093/bib/bbad074.

22. Sheng Y, Jiang Y, Yang Y, Li X, Qiu J, Wu J, et al. CNA2Subpathway: identification of dysregulated subpathway driven by copy number alterations in cancer. Brief Bioinform. 2021;22 5 doi:10.1093/bib/bbaa413.

23. Ciou HH, Lee TH, Wang HC, Ding YR, Tseng CJ, Wang PH, et al. Repurposing gestrinone for tumor suppressor through P21 reduction regulated by JNK in gynecological cancer. Transl Res. 2022;243:21-32. doi:10.1016/j.trsl.2021.12.002.

24. Lamb CA, Fabris VT and Lanari C. Progesterone and breast. Best Pract Res Clin Obstet Gynaecol. 2020;69:85-94. doi:10.1016/j.bpobgyn.2020.04.001.

25. Yip CH and Rhodes A. Estrogen and progesterone receptors in breast cancer. Future Oncol. 2014;10 14:2293-301. doi:10.2217/fon.14.110.

26. Aamdal S, Bormer O, Jorgensen O, Host H, Eliassen G, Kaalhus O, et al. Estrogen receptors and long-term prognosis in breast cancer. Cancer. 1984;53 11:2525-9. doi:10.1002/1097-0142(19840601)53:11<2525::aid-cncr2820531126>3.0.co;2-8.

27. Ashourpour M, Mostafavi Hosseini F, Amini M, Saeedian Moghadam E, Kazerouni F, Arman SY, et al. Pyrazole Derivatives Induce Apoptosis via ROS Generation in the Triple Negative Breast Cancer Cells, MDA-MB-468. Asian Pac J Cancer Prev. 2021;22 7:2079-87. doi:10.31557/APJCP.2021.22.7.2079.

28. Wen J, Bao Y, Niu Q, Yang J, Fan Y, Li J, et al. Identification of N-(6-mercaptohexyl)-3-(4-pyridyl)-1H-pyrazole-5-carboxamide and its disulfide prodrug as potent histone deacetylase inhibitors with in vitro and in vivo anti-tumor efficacy. Eur J Med Chem. 2016;109:350-9. doi:10.1016/j.ejmech.2016.01.013.

29. Moore NL, Hanson AR, Ebrahimie E, Hickey TE and Tilley WD. Anti-proliferative transcriptional effects of medroxyprogesterone acetate in estrogen receptor positive breast cancer cells are predominantly mediated by the progesterone receptor. J Steroid Biochem Mol Biol. 2020;199:105548. doi:10.1016/j.jsbmb.2019.105548.

30. Raje N, Kumar S, Hideshima T, Roccaro A, Ishitsuka K, Yasui H, et al. Seliciclib (CYC202 or R-roscovitine), a small-molecule cyclin-dependent kinase inhibitor, mediates activity via down-regulation of Mcl-1 in multiple myeloma. Blood. 2005;106 3:1042-7. doi:10.1182/blood-2005-01-0320.

31. Schutte B, Nieland L, van Engeland M, Henfling ME, Meijer L and Ramaekers FC. The effect of the cyclin-dependent kinase inhibitor olomoucine on cell cycle kinetics. Exp Cell Res. 1997;236 1:4-15. doi:10.1006/excr.1997.3700.

32. Abraham RT, Acquarone M, Andersen A, Asensi A, Belle R, Berger F, et al. Cellular effects of olomoucine, an inhibitor of cyclin-dependent kinases. Biol Cell. 1995;83 2-3:105-20. doi:10.1016/0248-4900(96)81298-6.

33. Arai K, Eguchi T, Rahman MM, Sakamoto R, Masuda N, Nakatsura T, et al. A Novel High-Throughput 3D Screening System for EMT Inhibitors: A Pilot Screening Discovered the EMT Inhibitory Activity of CDK2 Inhibitor SU9516. PLoS One. 2016;11 9:e0162394. doi:10.1371/journal.pone.0162394.

34. Paliouras AR, Buzzetti M, Shi L, Donaldson IJ, Magee P, Sahoo S, et al. Vulnerability of drug-resistant EML4-ALK rearranged lung cancer to transcriptional inhibition. EMBO Mol Med. 2020;12 7:e11099. doi:10.15252/emmm.201911099.

35. Shapiro GI. Preclinical and clinical development of the cyclin-dependent kinase inhibitor flavopiridol. Clin Cancer Res. 2004;10 12 Pt 2:4270s-5s. doi:10.1158/1078-0432.CCR-040020.

36. Oprea TI and Overington JP. Computational and Practical Aspects of Drug Repositioning. Assay Drug Dev Technol. 2015;13 6:299-306. doi:10.1089/adt.2015.29011.tiodrrr.

Figures legends

Figure 1. Schematic overview of the DrugSim2DR method.

Figure 2. Assessment of drug-drug functional similarity in the context of breast cancer. (A-B) Comparations of chemical structure similarity and semantic-based similarity across four groups (Q1-Q4) were categorized according to the quartiles of the functional similarity scores. Statistical significance between box plots is calculated by Wilcoxon rank-sum tests (****, p-value < 0.0001). (C-E) Bipartite networks of drugs and their shared GO terms for ouabain/tegoprazan, ouabain/bisacodyl, and bisacodyl/tegoprazan pairs. The red nodes in the network represent drugs and the blue ones represent GO terms.

Figure 3. Heatmap of gene expression levels of drugs' targets between breast cancer and normal samples.

Figure 4. Performance of the DrugSim2DR approach. (A) ROC curves for drugs identified by DrugSim2DR in five different cancer types. The AUROC values for drugs in each cancer type are calculated and displayed respectively. (B) Comparison of DrugSim2DR with three other approaches. We apply DrugSim2DR to three cancer types to compare the performance with the CMap, SubtypeDrug and DvD. AUROC values are used to compare their performance.

Figure 5. Robustness analysis of DrugSim2DR on breast cancer dataset. (A) Radar chart showing the overlapped number of top 50 drugs identified based on the restart probability r values set from 0.1 to 0.8 compared with that of r=0.9. (B) Venn diagram showing the overlapped number of the top 50 drugs identified in the GSE53752, GSE42568, and GSE21422 datasets. (C) Boxplots showing the AUROC values of predicted drugs for the different data removal. The red line indicates the AUROC value of the original data.

Table 1. Top 10 functionally similar drug pairs in the context of breast cancer.

Drug 1	Drug 2	Drug Name 1	Drug Name 2	Similarity Score
DB01213	DB02721	Fomepizole	4-Iodopyrazole	17.27
DB02721	DB02757	4-Iodopyrazole	Pyrazole	5.75
DB01213	DB02757	Fomepizole	Pyrazole	5.13
DB00562	DB00819	Benzthiazide	Acetazolamide	4.18
DB01092	DB16690	Ouabain	Tegoprazan	4.09
DB01092	DB09020	Ouabain	Bisacodyl	3.80
DB00562	DB08846	Benzthiazide	Ellagic acid	3.52
DB00562	DB00606	Benzthiazide	Cyclothiazide	3.46
DB09020	DB16690	Bisacodyl	Tegoprazan	3.32
DB00819	DB08846	Acetazolamide	Ellagic acid	3.31

Table 2. Candidate drugs for breast cancer identified by DrugSim2DR with FDR < 0.1.

DrugBank ID	Drug Name	Main indications	Centrality Score	FDR	Evidence
DB01185	Fluoxymesterone	Breast cancer/ Hypogonadism	0.0051	< 0.001	FDA approved
DB11619	Gestrinone	Endometriosis	0.0050	< 0.001	PMID: 34921996
DB02757	Pyrazole		0.0045	< 0.001	PMID: 34319030
DB01213	Fomepizole	Ethylene glycol poisoning	0.0026	< 0.001	
DB00603	Medroxyprogesterone acetate	Metastatic Renal Cell Carcinoma/ Metastatic Endometrial carcinoma	0.0025	< 0.001	PMID: 31805393

Click here to access/download;Figure;Figure 1.tif

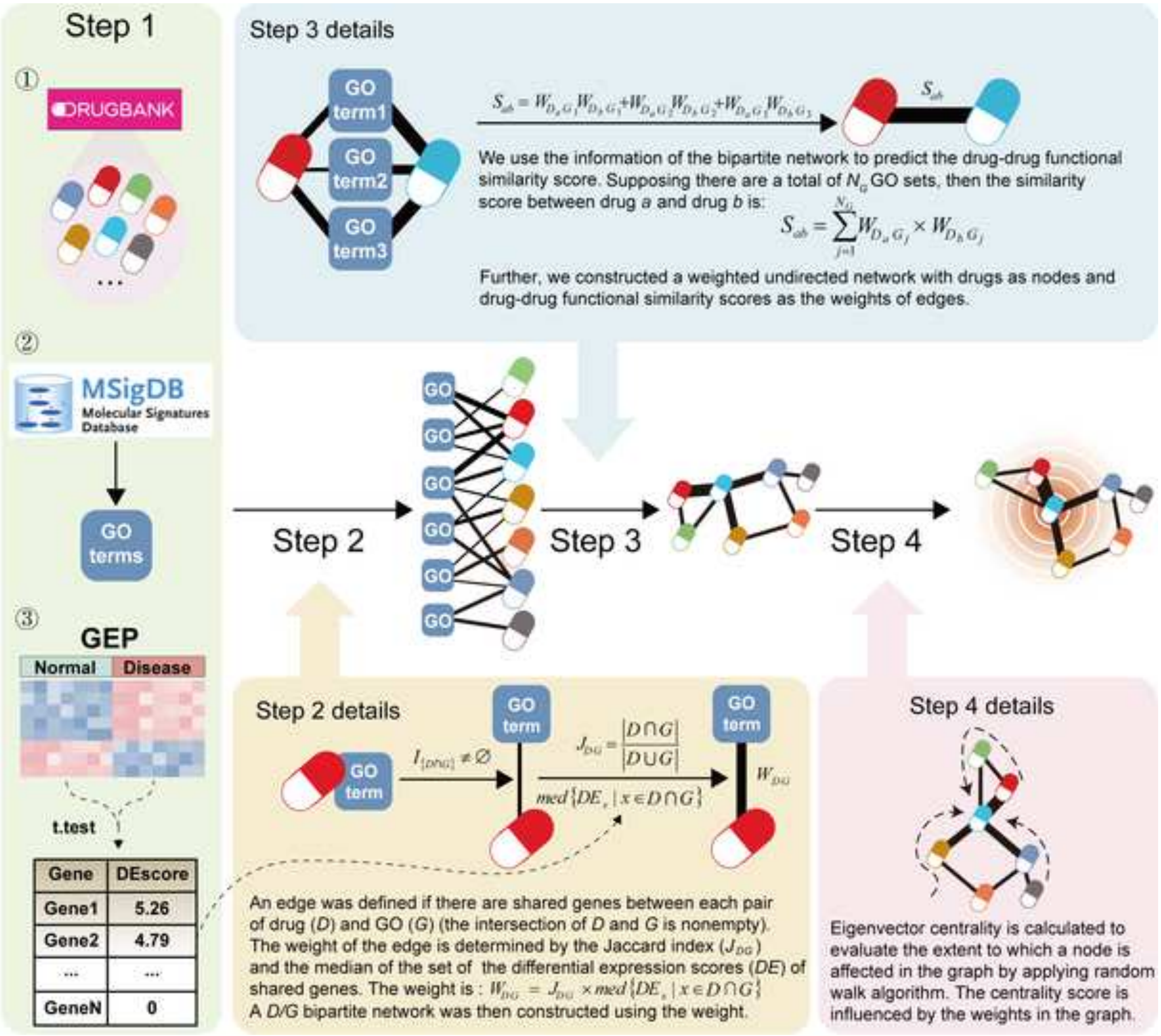

Click here to access/download;Figure;Figure 2.tif

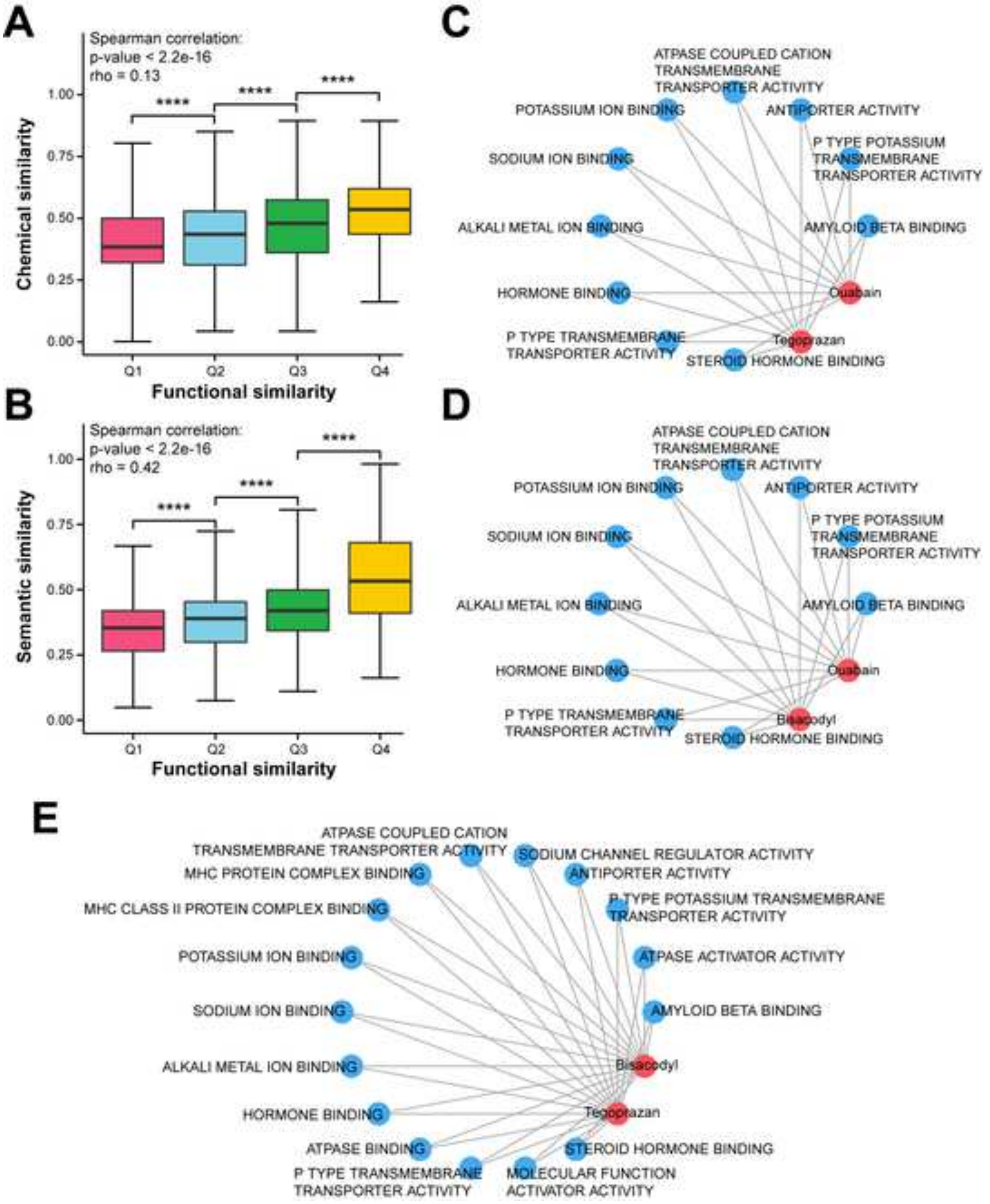

Click here to access/download;Figure;Figure 3.tif

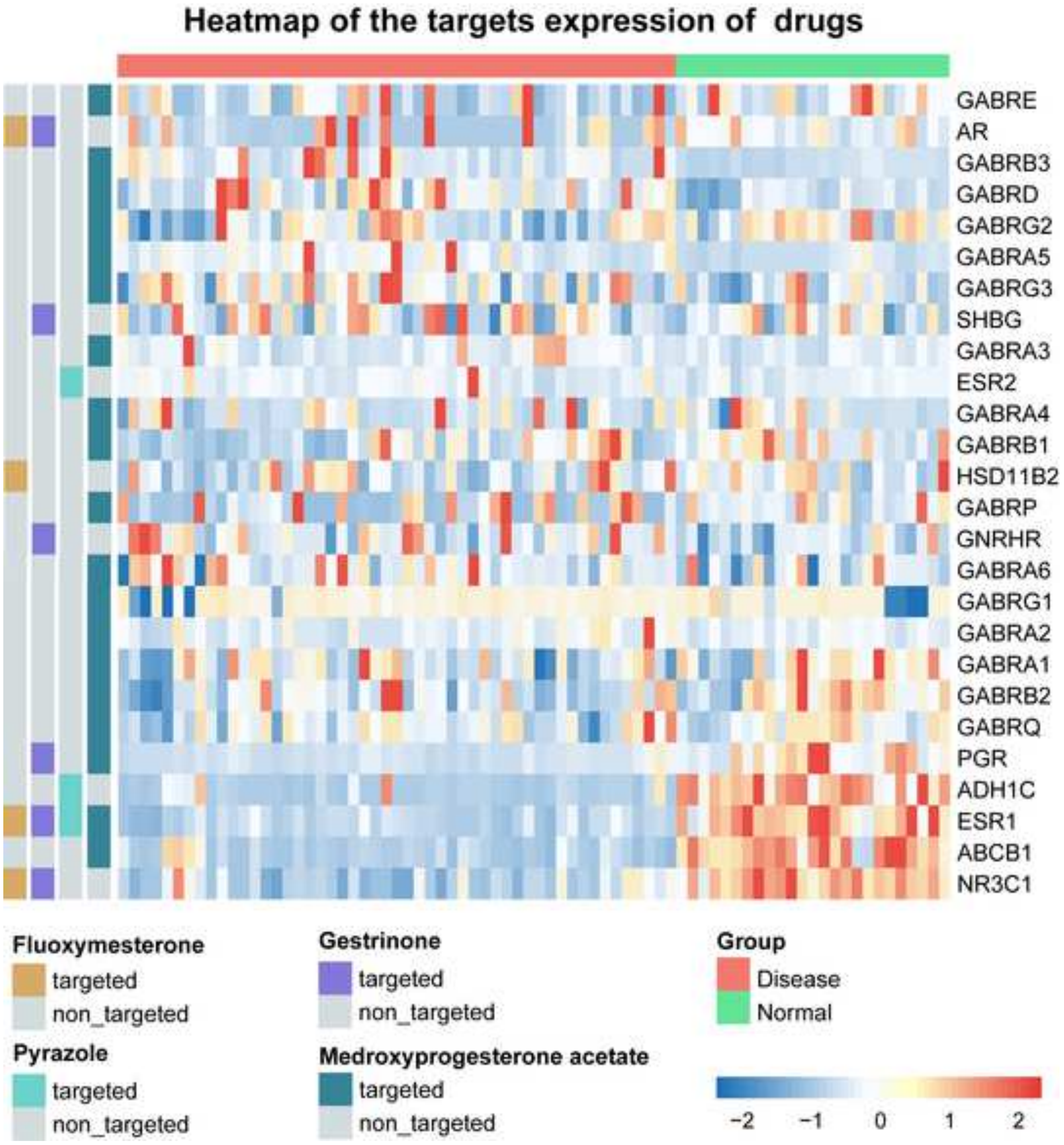

Click here to access/download;Figure;Figure 4.tif

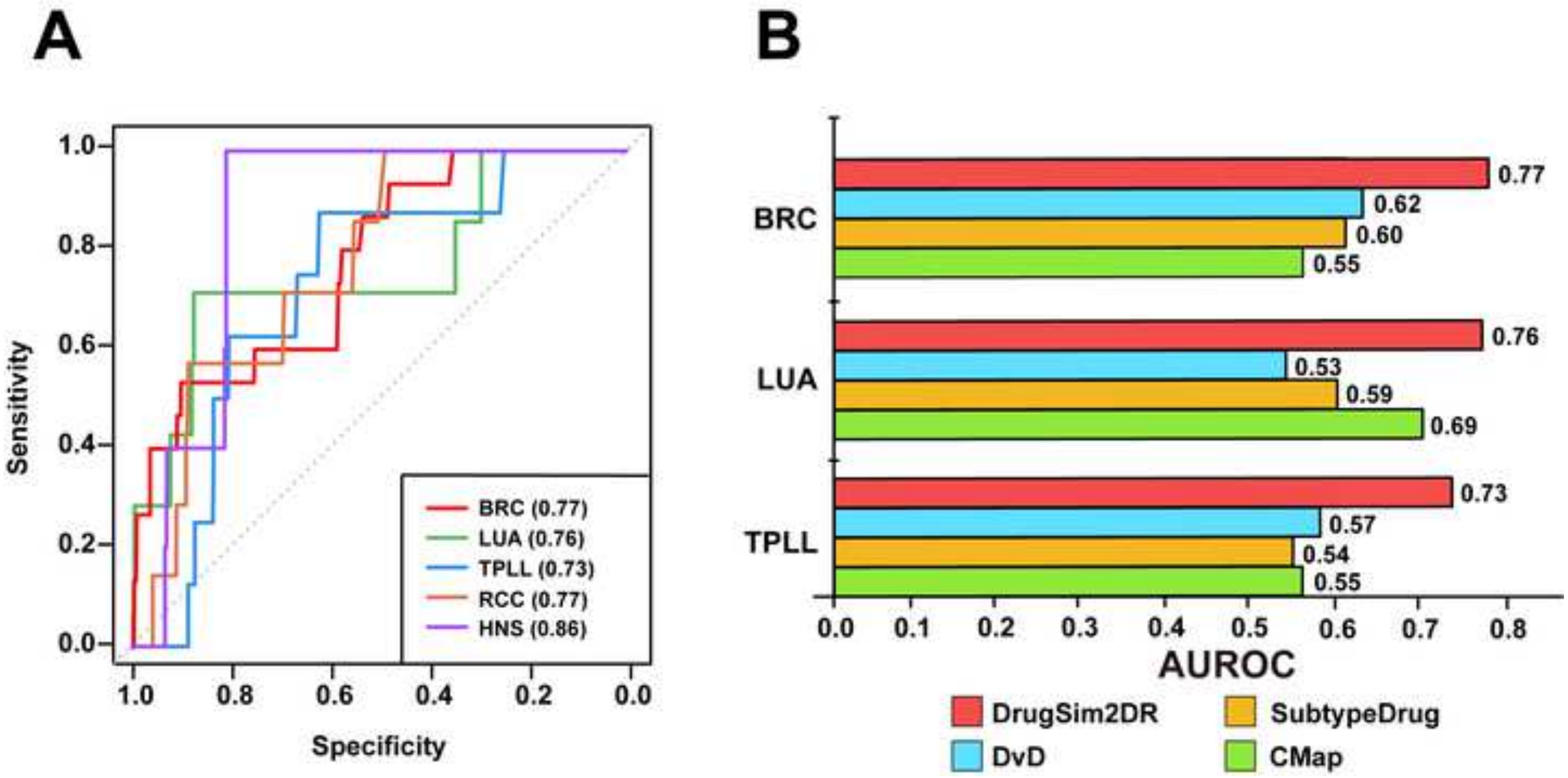

Click here to access/download;Figure;Figure 5.tif

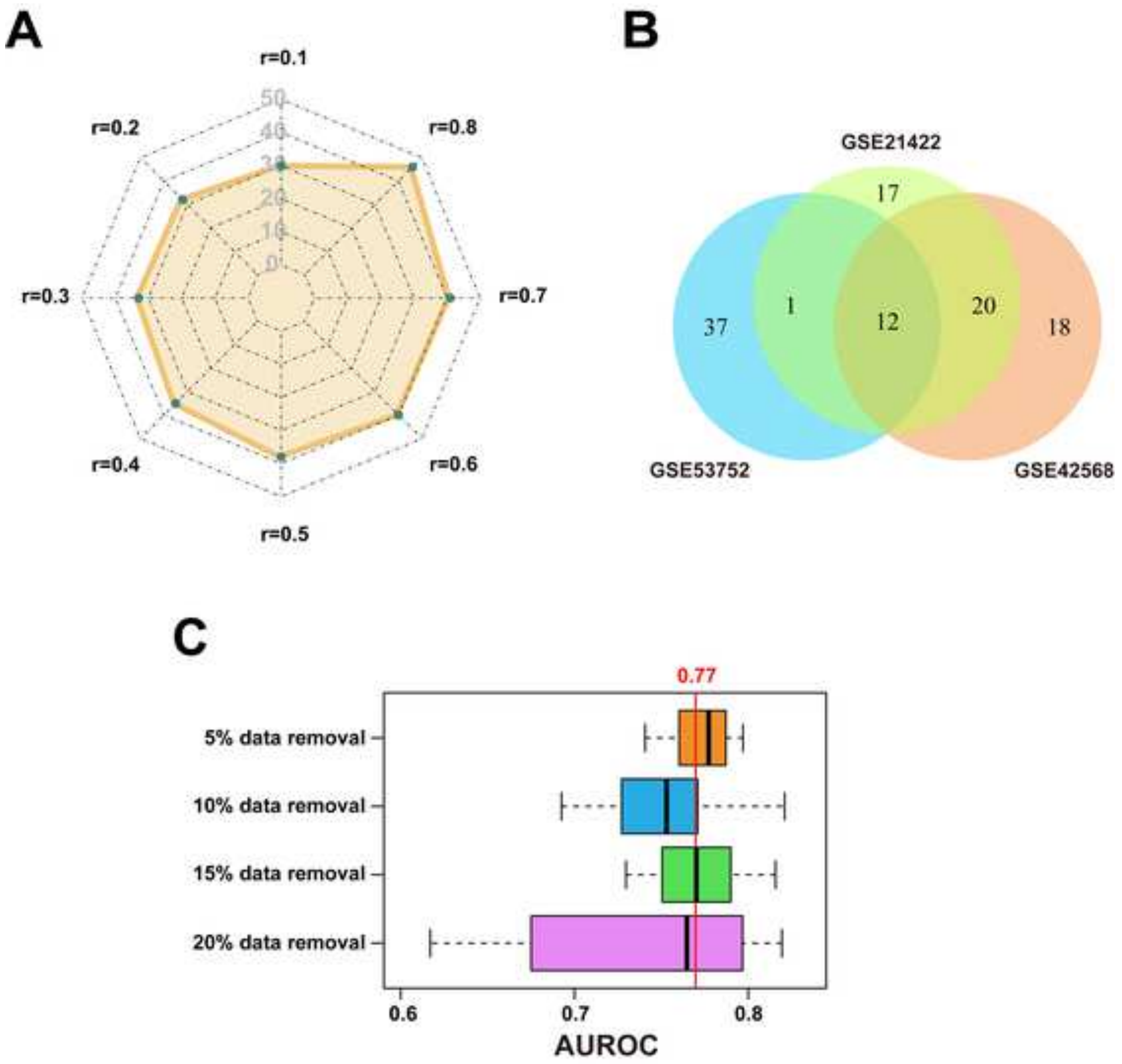

Click here to access/download
Supplementary Material
Wu J et al Supplement material.pdf

Table

Click here to access/download
Supplementary Material
Table S1_Supplementary Material.xlsx

Click here to access/download
Supplementary Material
Figure S1_Supplementary Material.tif

Click here to access/download
Supplementary Material
Figure S2_Supplementary Material.tif

Click here to access/download
Supplementary Material
Figure S3_Supplementary Material.tif
