# Supplementary material for: DrugSim2DR: systematic prediction of drug functional similarities in the context of specific disease for drug repurposing
Source: Gigascience. 2023 Dec 19;12:giad104. doi: 10.1093/gigascience/giad104 (PMC10729734; doi:10.1093/gigascience/giad104)
Supplement: giad104_Supplemental_Files [file giad104_supplemental_files.zip › Wu J et al Supplement material.pdf]

Supplementary Materials for
DrugSim2DR: Systematic prediction of drug functional similarities in the context of specific disease for drug repurposing

This PDF file includes:
Supplementary Text 1
Supplementary Figure S1-3

Supplementary Text 1

Chemical structure-based drug-drug similarity

Here, we aimed to calculate the chemical structure-based drug-drug similarity score. To characterize the chemical structure, we downloaded the simplified molecular input line entry system (SMILES) of all drug molecules from the ChEMBL database (https://www.ebi.ac.uk/chembl/). We used R "rcdk" package to convert the SMILES of each drug into a Morgan fingerprint (1024bit), which is a binary vector consisting of "0" and "1", indicating the absence or presence of a specific substructure. The Tanimoto coefficient (TC) was calculated based on the fingerprints of two drugs to measure their level of chemical structural similarity. The TC_{ab} between the fingerprints of drug *a* and drug *b* is calculated as follows:

$$TC_{ab} = \frac{k}{(n + m - k)} \qquad (1)$$

where *n* and *m* are the count of "1" in the fingerprint of drug *a* and *b* respectively; *k* is the count of bits that are represented as "1" in both fingerprints simultaneously. Tanimoto similarity has proven to be a reliable option for determining the similarity between fingerprints [1].

Semantic-based drug-drug similarity

To measure the semantic-based similarity between any two drugs, we first used a graph-based semantic similarity algorithm to determine the semantic similarity between the drug-associated GO terms (the intersection between a GO term and a set of drug targets is non-empty). The algorithm was implemented through "mgosim" function in R "GOSemSim" package [2]. Then, pairwise semantic similarities between any two GO terms associated with drug *a* and drug *b* were averaged into the drug-drug semantic-based similarity S_{ab}:

$$S_{ab} = \frac{1}{n \times m} \sum_{i=1}^{n} \sum_{j=1}^{m} mgosim(G_i^a, G_j^b) \qquad (2)$$

where G^a and G^b are the GO terms associated with drug *a* and *b* respectively; *n* and *m* are the count of G^a and G^b.

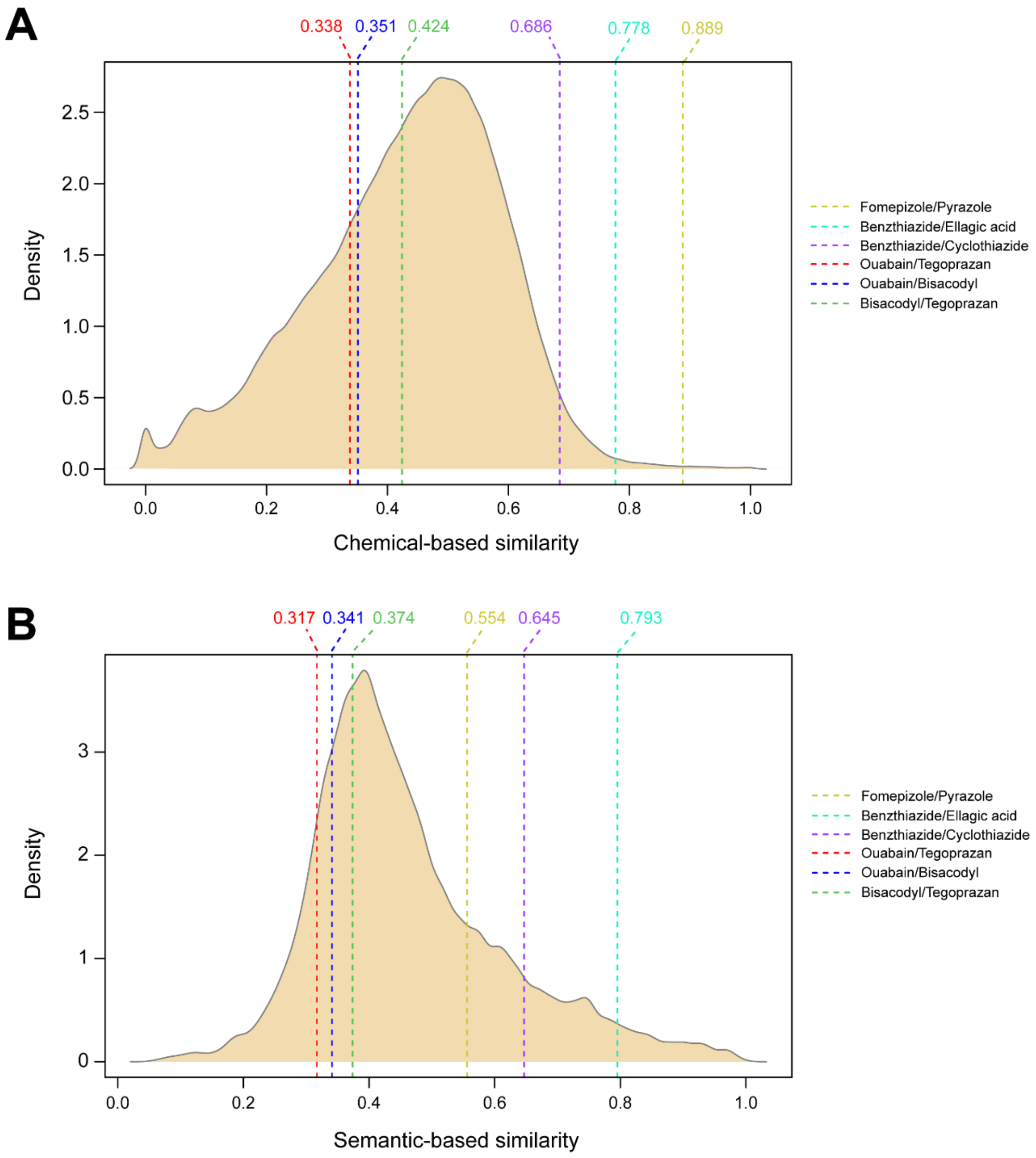

Supplementary Figure S1. Kernel density curve of chemical structure- and semantic-based drug-drug similarity scores. **(A)** Density curve of chemical-based similarity scores. **(B)** Density curve of semantic-based similarity scores. Several drug pairs with high functional similarity in the context of breast cancer were assigned to the curves respectively and represented by distinct colors

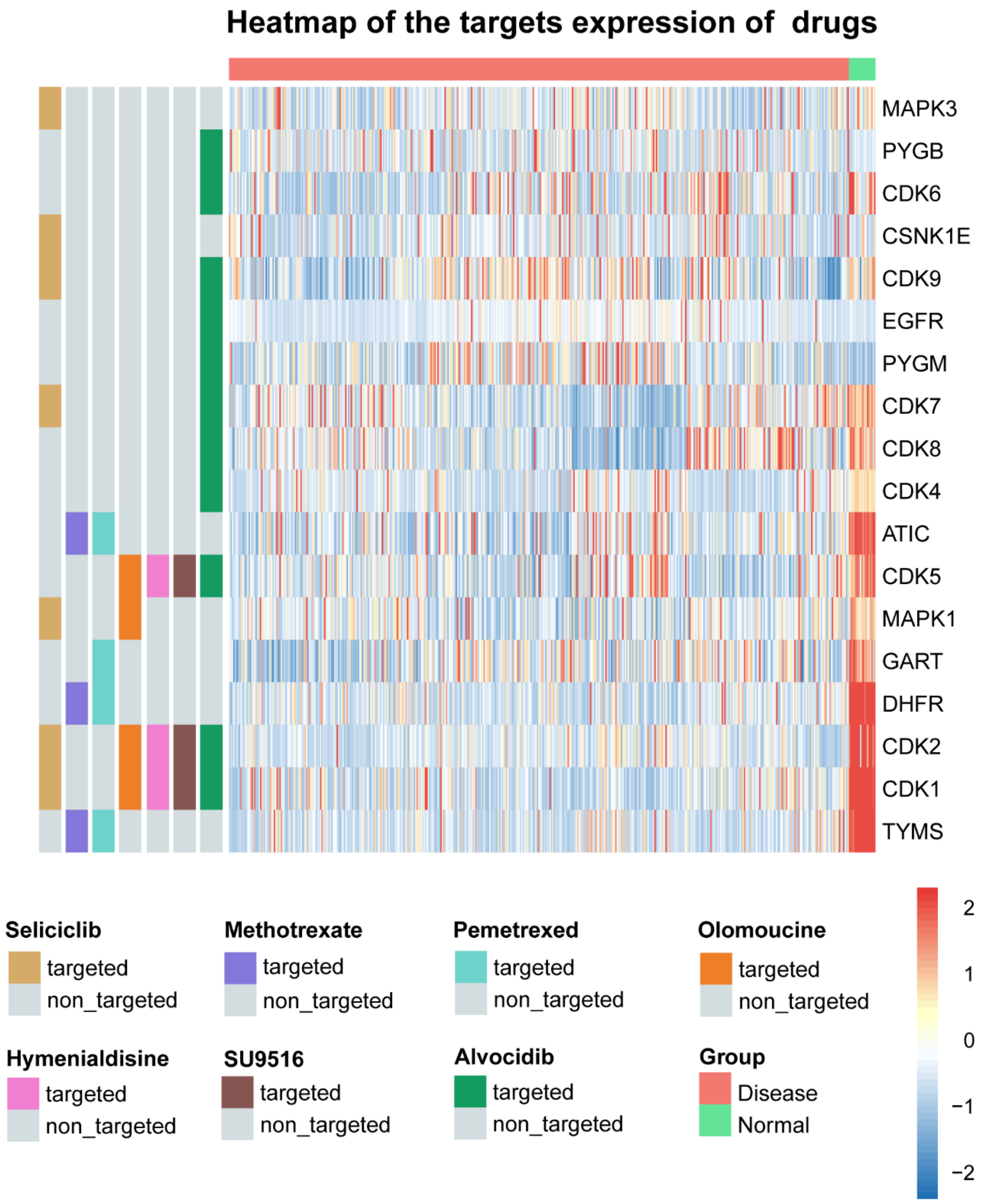

Supplementary Figure S2. Heatmap of gene expression levels of drugs' targets between lung cancer and normal samples.

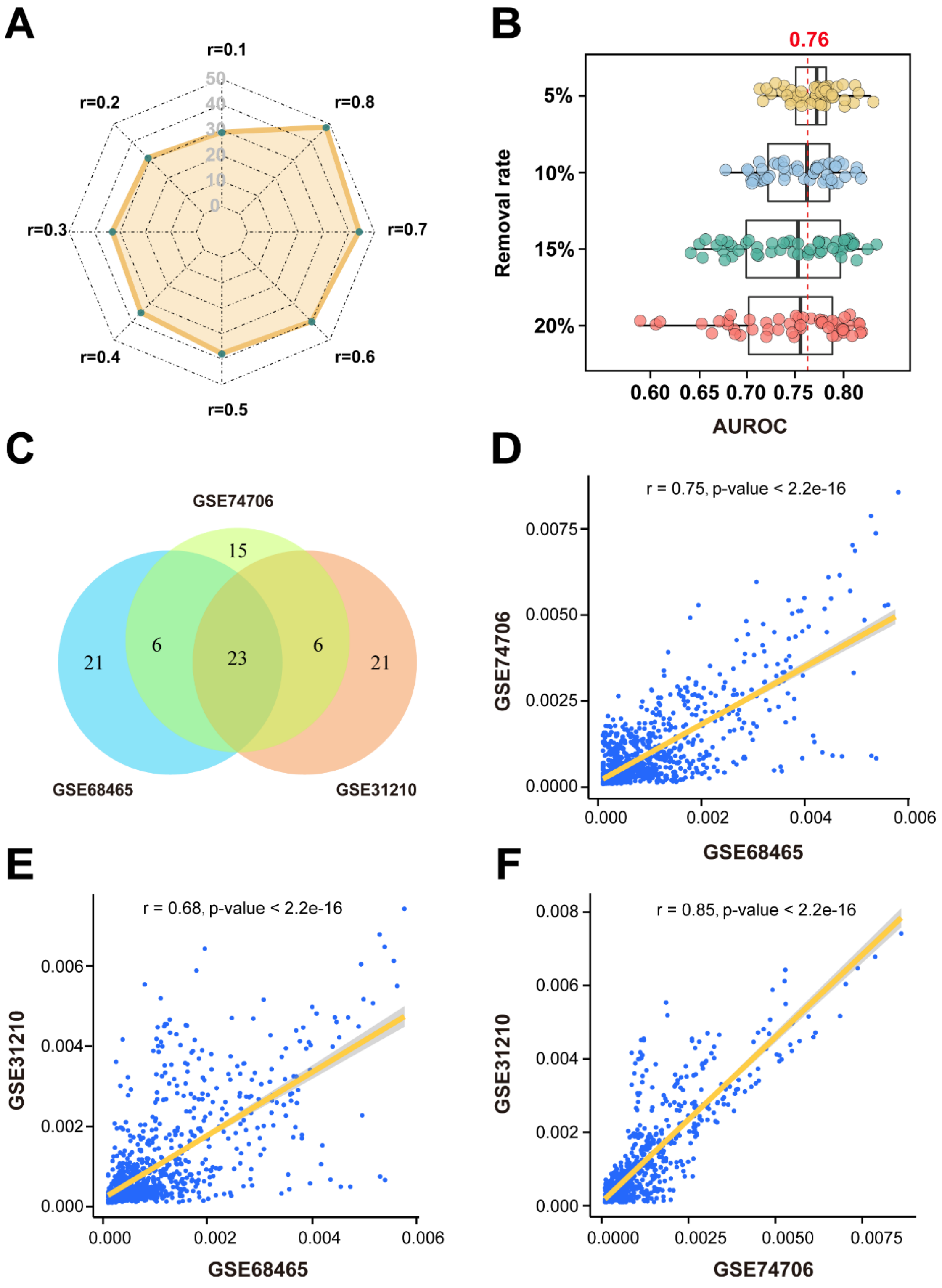

Supplementary Figure S3. Robustness and reproducibility analysis of DrugSim2DR on lung cancer dataset. **(A)** Radar chart showing the overlapped number of top 50 drugs identified based on the restart probability r values set from 0.1 to 0.8 compared with that of r=0.9. **(B)** Boxplots showing the AUC values of predicted drugs for the

different data removal. The red line indicates the AUC value of the original data. **(C)** Venn diagram showing the overlapped number of the top 50 drugs identified in the GSE68465, GSE74706, and GSE31210 datasets. Correlation analysis of DrugSim2DR's predictions for lung cancer across different datasets: **(D)** GSE68465 and GSE74706; **(E)** GSE68465 and GSE31210; **(F)** GSE74706 and GSE31210.

References

1. Bajusz D, Racz A and Heberger K. Why is Tanimoto index an appropriate choice for fingerprint-based similarity calculations? J Cheminform. 2015;7:20. doi:10.1186/s13321-015-0069-3.
2. Yu G, Li F, Qin Y, Bo X, Wu Y and Wang S. GOSemSim: an R package for measuring semantic similarity among GO terms and gene products. Bioinformatics. 2010;26 7:976-8. doi:10.1093/bioinformatics/btq064.
