# Supplementary material for: DrugSim2DR: systematic prediction of drug functional similarities in the context of specific disease for drug repurposing
Source: Gigascience. 2023 Dec 19;12:giad104. doi: 10.1093/gigascience/giad104 (PMC10729734; doi:10.1093/gigascience/giad104)
Supplement: giad104_Response_to_Reviewer_Comments_Original_Submission [file giad104_response_to_reviewer_comments_original_submission.pdf]

Author's Response To Reviewer Comments

Dear Prof. Hongling Zhou and reviewers:

Thank you very much for your letter and for the reviewers' comments concerning our manuscript entitled "DrugSim2DR: Systematic prediction of drug functional similarities in the context of specific disease for drug repurposing" (Manuscript ID: GIGA-D-23-00219). Those comments are all valuable and very helpful for revising and improving our paper, as well as the important guiding significance to our research. We have studied comments carefully and have made corrections which we hope meet with approval. If you or the reviewers have any other suggestions, we are willing to revise them until you are satisfied. Revised portions are marked in RED in the paper. The main corrections in the paper and the responses to the reviewer's comments are as following:

Reply to Editor
Please register any new software application in the bio.tools and SciCrunch.org databases to receive RRID (Research Resource Identification Initiative ID) and biotoolsID identifiers, and include these in your manuscript. Computational workflows should be registered in workflowhub.eu and the DOIs cited in the relevant places in the manuscript. These will facilitate tracking, reproducibility and re-use of your tool.

Please ensure you describe additional experiments that were carried out and include a detailed rebuttal of any criticisms or requested revisions that you disagreed with. Please also ensure that your revised manuscript conforms to the journal style, which can be found in the Instructions for Authors on the journal homepage. If the data and code has been modified in the revision process please be sure to update the public versions of this too.

Response: Thank you very much for your editorial efforts regarding our manuscript and for your recommendation that we submit a revision of this manuscript. In response to your request, we have registered our software on the bio.tools and SciCrunch.org databases and added RRID and biotoolsID identifiers to the section "Availability of Source Code and Requirements" in main manuscript. We have provided detailed responses to all the reviewers' comments. We can confirm that modifications have been made to the main manuscript and supplementary data, and additional experimental results have been included as required. Lastly, we have adjusted the overall style of the manuscript to ensure compliance with the journal's "Author Guidelines" section. We believe that the manuscript has been greatly strengthened by the critique of the reviewers and hope that both you and the reviewers will now find the paper suitable for publication.

Reply to Reviewer #1
Comments to the Author
In this paper, the authors developed a novel software package, named DrugSim2DR, to prioritize cancer-candidate drugs based on a weighted drug-drug functional similarity network constructed with the transcriptionally altered genes in the context of the specific disease state. They applied the method to different cancer types to show the predictive power and compare their results with other methods. I tried their software package and found it usable. Totally, this is a potentially interesting and publishable study. However, I have several concerns about this study.

Response: Thank you very much for your comments concerning our manuscript. These comments are valuable and insightful to improve our work. According to your comments, we have comprehensively revised the manuscript.

Point 1: The author chose drugs for network construction based on the count of target genes, with a range from 3 to 500. Might this result in excessively high degrees in certain nodes, thereby introducing bias into the network?

Response 1: Thank you for your invaluable comment. In our approach, drugs are initially linked to

molecular functions through target genes and subsequently connected to other drugs based on relevant molecular functions, and eventually form a drug network. As you correctly highlighted, the number of target genes is a critical factor. An excessive number of target genes for certain drugs can result in disproportionately high degrees of these drug nodes in the drug-drug network, thereby significantly overestimating their importance within the network. However, in our study, we construct the drug-drug network as a weighted network within the context of a specific disease. The importance of nodes determined by the random walk algorithm depends on the node's degree, the weights of connected edges, and the importance of neighboring nodes. Therefore, some nodes with only higher degrees won't lead to bias in the network. Furthermore, narrowing the selection of target genes would entail the removal of more drug data, thereby limiting the applicability of our approach. Consequently, we believe that the current target gene selection approach is appropriate and does not introduce network bias. We have added the relevant explanation to the main manuscript as follows.

Page 5-6, section "Data acquisition and processing" in the main manuscript.
"We selectively retained drugs possessing a target gene count of over 3 but fewer than 500 and obtained 1289 drugs. This screening strategy is deemed appropriate as it avoids the introduction of network bias and ensures maximal inclusion of drug data."

Point 2: In this study, the authors employed molecular function from Gene Ontology as an intermediary for evaluating drug-drug similarity. Why wasn't the biological process selected instead?

Response 2: I would like to express my gratitude for your comment. In our study, we utilized molecular function (MF) terms from Gene Ontology (GO) as a medium to establish relationships between drugs for drug repurposing. The reason we did not employ GO biological process (BP) is that, compared to GOMF, BP typically involves broader and more complex biological processes, which may encompass combinations of multiple molecular functions. Consequently, an analysis based on GOBP might be influenced by the complexity, making it challenging to capture subtle similarities or differences between drugs. We believe that employing GOMF allows us to attain a heightened precision in comprehending the interplay between drugs and biomolecules, consequently unveiling the mechanisms of drug action. This is crucial for comprehending the functions of drugs and for accurately assessing the similarity between them. Therefore, GOMF allows us to finely distinguish the similarities and dissimilarities between drugs without considering the intricacies of their actions in biological processes. This provides significant support for accurate drug repurposing in the subsequent steps. Hence, we opted to evaluate drug functional similarity through GOMF. We have added the relevant explanation to the main manuscript as follows.

Page 6, section "Data acquisition and processing" in the main manuscript.
"Then, Molecular Function (MF) gene sets derived from Gene Ontology (GO) were downloaded from the Molecular Signature Database (https://www.gsea-msigdb.org/gsea/msigdb/index.jsp, version 7.5.1). We believe that employing GOMF allows us to attain a heightened precision in comprehending the interplay between drugs and biomolecules, consequently unveiling the mechanisms of drug action. This is crucial for comprehending the functions of drugs and for accurately assessing the similarity between them."

Point 3: When evaluating drug repositioning performance compared to other methodologies, it is advisable to incorporate more detailed result comparisons. For instance, comparing the top ten candidate drugs identified by various methods, beyond solely relying on AUROC comparisons, can provide a more comprehensive assessment.

Response 3: Thank you very much for your suggestions. As you mentioned, a detailed comparison of results can enhance the explanation of method performance. Given that DrugSim2DR and other approaches use different background drugs, we compared the number of evidence-supported drugs among the top n drugs identified by different approaches, where n is equal to the number of candidate drugs identified by DrugSim2DR according to FDR < 0.1. We believe that this comparison not only strengthens the explanation of the results but also ensures fairness in the evaluation. The final results indicate that DrugSim2DR can identify the highest number of evidence-supported candidate drugs for different diseases. This further underscores the excellent predictive capability of our approach.
We have added the relevant content to the main manuscript and included the results in the Supplementary files as Supplementary Table S2-4.

Page 16, section “Comparison of DrugSim2DR with other approaches” in the main manuscript.
“According to the ROC curve analysis, DrugSim2DR showed better AUROC values than the other approaches in these datasets (Figure 4B). In addition to AUROC, it is crucial that candidate drugs have sufficient research support for their disease-inhibiting capabilities. Therefore, we compared the number of evidence-supported drugs among the top n drugs identified by different approaches and DrugSim2DR, where n is equal to the number of candidate drugs identified by DrugSim2DR. As shown in Supplementary Table S2-4, DrugSim2DR can identify the highest number of evidence-supported candidate drugs for different diseases. This further underscores the excellent predictive capability of our approach.”

Page 21, section “Additional Files” in the main manuscript.
“Supplementary Table S2-4. Comparison of candidate drugs identified by different approaches.”

Point 4: The author highlights method reproducibility by comparing the top 50 drugs across diverse datasets. However, I recommend extending the assessment of result consistency beyond the top-ranked drugs and including correlation analysis for a comprehensive view of drug optimization list consistency.

Response 4: We sincerely appreciate your valuable suggestions. As you mentioned, our previous validation of the approach's robustness was not comprehensive. In response to your guidance, we conducted correlation analyses on the drug score lists optimized by our approach across distinct datasets related to the same disease, thus fortifying the evidence of reproducibility. In detail, we initially applied DrugSim2DR to three separate breast cancer datasets, subjecting each pair of optimized result lists to Pearson correlation tests. The outcomes unveiled pairwise statistically significant high correlations. Similar results were achieved in the context of lung cancer. These findings provide further substantiation for the robust reproducibility of DrugSim2DR.
We have incorporated the additional content into the main manuscript and accordingly added the resulting figures as Figure 5D-F and Supplementary Figure S3D-F.

Page 17-18, section “Reproducibility analysis of DrugSim2DR” in the main manuscript.
“To provide a general comparison, the top 50 drugs for each ranked drug list were used. As shown in Figure 5C, 12 overlapped drugs were found among the three datasets. To further demonstrate the reproducibility of DrugSim2DR, we chose to perform a comprehensive consistency validation of the results. Specifically, we conducted correlation analysis on the three entire drug centrality score lists. The outcomes unveiled pairwise statistically significant high correlations (Figure 5D-F, Pearson correlation test p-value < 2.2e-16). In the same way, we also performed the reproducibility validation on three LUA datasets: GSE68465, GSE74706 and GSE31210. Supplementary Figure S3C exhibited that the overlapping ratios of drugs in these three lung cancers are 46% (23/50). The drug centrality scores optimized by DrugSim2DR in different lung cancer datasets still exhibit a strong correlation (Supplementary Figure S3D-F, Pearson correlation test p-value < 2.2e-16). These results demonstrate the reproducibility of our approach for the different datasets of the same disease.”

Page 27, section “Figures legends” in the main manuscript.
“Figure 5. Robustness and reproducibility analysis of DrugSim2DR on breast cancer dataset. (A) Radar chart showing the overlapped number of top 50 drugs identified based on the restart probability r values set from 0.1 to 0.8 compared with that of r=0.9. (B) Boxplots showing the AUROC values of predicted drugs for the different data removal. The red line indicates the AUROC value of the original data. (C) Venn diagram showing the overlapped number of the top 50 drugs identified in the GSE53752, GSE42568, and GSE21422 datasets. Correlation analysis of DrugSim2DR's predictions for breast cancer across different datasets: (D) GSE53752 and GSE42568; (E) GSE53752 and GSE21422; (F) GSE42568 and GSE21422.”

Page 3, section “Supplementary Figure Legends” in the Supplementary Materials.
“Supplementary Figure S3. Robustness and reproducibility analysis of DrugSim2DR on lung cancer dataset. (A) Radar chart showing the overlapped number of top 50 drugs identified based on the restart probability r values set from 0.1 to 0.8 compared with that of r=0.9. (B) Boxplots showing the AUC values of predicted drugs for the different data removal. The red line indicates the AUC value of the original data. (C) Venn diagram showing the overlapped number of the top 50 drugs identified in the GSE68465, GSE74706, and GSE31210 datasets. Correlation analysis of DrugSim2DR's predictions for breast cancer across different

datasets: (D) GSE68465 and GSE74706; (E) GSE68465 and GSE31210; (F) GSE74706 and GSE31210."

Point 5: In the section titled "Robustness Analysis of DrugSim2DR," the validation of the method's robustness and reproducibility is conducted. I recommend that the author dedicate a separate paragraph to the aspects related to reproducibility to enhance the clarity of the article's structure.

Response 5: We greatly appreciate your guidance regarding our writing. In accordance with your suggestions, we have separated the content related to reproducibility validation from the "Robustness analysis of DrugSim2DR" thereby establishing it as an independent "Reproducibility analysis of DrugSim2DR" section. Furthermore, we have meticulously restructured both the logical flow and the overall organization of the article to enhance its clarity and coherence. Once again, we extend our gratitude for your valuable input.

Page 17, section "Robustness analysis of DrugSim2DR" in the main manuscript.
"The result for the BRC dataset showed that the percentages of overlapped drugs increased with r slowly and all above 60% (30/50) for the different r values (Figure 5A), proving the robustness of DrugSim2DR's results to changes in the restart probability. For the LUA dataset, we obtained similar results (Supplementary Figure S3A)."

"To further assess the robustness of the DrugSim2DR, we performed the data removal tests using the BRC and LUA datasets. We removed the gene expression values from 5% to 20% at 5% intervals and reapplied the DrugSim2DR approach 50 times for each removal. For each removal, we performed the ROC analysis and calculated the AUROC values. The results showed that the different data removals had only a weak effect on the accuracy of the predictions. The median AUROC value remained above 0.75, even after the removal of up to 20% of the gene expression data in the BRC and LUA datasets (Figure 5B and Supplementary Figure S3B), indicating that the DrugSim2DR approach is robust to data removal."

Page 17-18, section "Reproducibility analysis of DrugSim2DR" in the main manuscript.
"Reproducibility analysis of DrugSim2DR. Due to the presence of intra-tumor heterogeneity and the impact of different platforms on sequencing results, the reproducibility of the approach's results in different datasets of the same disease is critical. To evaluate the reproducibility of DrugSim2DR, we downloaded another two BRC gene expression datasets from the GEO database: GSE42568 and GSE21422. We applied DrugSim2DR to these datasets and compared their results with that of the initial GSE53752 dataset. To provide a general comparison, the top 50 drugs for each ranked drug list were used. As shown in Figure 5C, 12 overlapped drugs were found among the three datasets. To further demonstrate the reproducibility of DrugSim2DR, we chose to perform a comprehensive consistency validation of the results. Specifically, we conducted correlation analysis on the three entire drug centrality score lists. The outcomes unveiled pairwise statistically significant high correlations (Figure 5D-F, Pearson correlation test p-value < 2.2e-16). In the same way, we also performed the reproducibility validation on three LUA datasets: GSE68465, GSE74706 and GSE31210. Supplementary Figure S3C exhibited that the overlapping ratios of drugs in these three lung cancers are 46% (23/50). The drug centrality scores optimized by DrugSim2DR in different lung cancer datasets still exhibit a strong correlation (Supplementary Figure S3D-F, Pearson correlation test p-value < 2.2e-16). These results demonstrate the reproducibility of our approach for the different datasets of the same disease."

Page 27, section "Figures legends" in the main manuscript.
"Figure 5. Robustness and reproducibility analysis of DrugSim2DR on breast cancer dataset. (A) Radar chart showing the overlapped number of top 50 drugs identified based on the restart probability r values set from 0.1 to 0.8 compared with that of r=0.9. (B) Boxplots showing the AUROC values of predicted drugs for the different data removal. The red line indicates the AUROC value of the original data. (C) Venn diagram showing the overlapped number of the top 50 drugs identified in the GSE53752, GSE42568, and GSE21422 datasets. Correlation analysis of DrugSim2DR's predictions for breast cancer across different datasets: (D) GSE53752 and GSE42568; (E) GSE53752 and GSE21422; (F) GSE42568 and GSE21422."

Page 3, section "Supplementary Figure Legends" in the Supplementary Materials.
"Supplementary Figure S3. Robustness and reproducibility analysis of DrugSim2DR on lung cancer dataset. (A) Radar chart showing the overlapped number of top 50 drugs identified based on the restart probability

r values set from 0.1 to 0.8 compared with that of r=0.9. (B) Boxplots showing the AUC values of predicted drugs for the different data removal. The red line indicates the AUC value of the original data. (C) Venn diagram showing the overlapped number of the top 50 drugs identified in the GSE68465, GSE74706, and GSE31210 datasets. Correlation analysis of DrugSim2DR's predictions for breast cancer across different datasets: (D) GSE68465 and GSE74706; (E) GSE68465 and GSE31210; (F) GSE74706 and GSE31210."

Page 21, section "Additional Files" in the main manuscript.
"Supplementary Figure S3. Robustness and reproducibility analysis of DrugSim2DR on lung cancer dataset."

Point 6: There are some grammatical problems in the passage. For example, the "statistical significance" in the part "Prioritizing disease candidate drugs through a network propagation algorithm" should be changed to "statistically significant". Please check the description of inappropriate parts of the text and correct it.

Response 6: Thank you for pointing this out. According to your comments, we have revised the irregular writing and grammatical problems in the main manuscript as follows.

Page 8, section "Constructing a drug-drug functional similarity network" in the main manuscript.
"We consider that the functional similarity score between two drugs will be stronger when they share more neighbor GO terms."

Page 10, section "Prioritizing disease candidate drugs through a network propagation algorithm" in the main manuscript.
"As the intersection is constant, and the DE-scores of genes are variable, we used bootstrap resampling of our original DE-scores at the gene level, and repeated step 2-4 to find statistically significant candidate drugs."

Page 19, section "Discussion" in the main manuscript.
"Those drugs with significant centralities are reported as having potential therapeutic effects on the disease in their latent influence on the cascade of transcriptionally altered."

Point 7: I suggest using "where" to link equations (2) and (3), or alternatively, swapping their order to enhance reader comprehension.

Response 7: Thank you very much for your suggestions. Following your advice, we have reordered equations (2) and (3) in section "Constructing a Drug/GO bipartite network under the disease context" in the main manuscript. This adjustment provides readers with a more lucid and thorough comprehension of the equations' significance.

Reply to Reviewer #2
Comments to the Author
In this manuscript, the authors proposed a novel algorithm, named DrugSim2DR, to identify candidate anti-cancer drugs. The method used common biological functions as mediators to construct a drug-drug functional similarity network in a specific disease state to identify candidate anti-cancer drugs. The authors applied DrugSim2DR to breast and lung cancer datasets and achieved good predictive performance. Moreover, DrugSim2DR also showed higher AUROC values by comparing it with other methods. Overall, DrugSim2DR complemented existing drug repurposing methods, and its applicability is supported by a large amount of data and good results. However, some issues need to be revised before the manuscript is published.

REPLY: Your comment on our manuscript is greatly appreciated. Your insights have provided valuable guidance for the enhancement of our work. In line with your suggestions, we have undertaken comprehensive revisions to the manuscript. Thank you for your valuable input.

Major comments

Point 1: In the data processing section, the author delineated the drug selection process predicated on the count of target genes. I deem this preliminary processing to be essential. However, I did not come across an account of how the author handled molecular function data. If the author refrained from preprocessing it, kindly elucidate the rationale. Alternatively, kindly specify the strategy employed for processing.

Response 1: Thank you very much for your comment. As you mentioned, data preprocessing is a crucial step. Actually, we conducted preprocessing for both drug data and molecular function data. For the acquired molecular function gene sets, we retained sets with gene numbers greater than 5 and less than 100, resulting in a total of 1330 functional sets. This ensures that these molecular function sets maintain an appropriate balance between specificity and generality. We apologize for overlooking the description of the molecular function preprocessing strategy. We have now added a detailed explanation to the "Data acquisition and processing" section in the main manuscript. Thank you once again for your comment.

Page 6, section "Data acquisition and processing" in the main manuscript.
"Then, Molecular Function (MF) gene sets derived from Gene Ontology (GO) were downloaded from the Molecular Signature Database (https://www.gsea-msigdb.org/gsea/msigdb/index.jsp, version 7.5.1). We retained MF sets with gene numbers greater than 5 and less than 100, resulting in a total of 1330 MF sets. This will avoid overly narrow or broad functional sets."

Point 2: In the breast cancer case study, the authors identified 5 potential anti-breast cancer drugs, but only four of them are shown in Figure 3, and the authors should make a reasonable explanation. The same problem appeared in the lung cancer study.

Response 2: Thank you very much for your suggestions. In the case of breast cancer, DrugSim2DR identified an FDA-approved anti-breast cancer drug and four candidate drugs according to FDR < 0.1. These candidate drugs have also been confirmed in the literature for their potential to inhibit the development of breast cancer. In Figure 3, we represented the aberrant expression of target genes for the candidate drugs through a heatmap. The aberration suggests that these candidate drugs may potentially intervene in the disease process by modulating the expression of their target genes, making them meaningful therapeutic options. As for FDA-approved drugs, we believe there is no need to explain their therapeutic potential through this approach. Therefore, in the heatmap, we displayed the target genes of only the four drugs. A similar situation exists in the case of lung cancer. We apologize for the lack of detailed explanations in the main manuscript. Following your advice, we have added appropriate explanations in the relevant sections of the main manuscript.

Page 14, section "Drug repurposing for breast cancer" in the main manuscript.
"Additionally, medroxyprogesterone acetate acts as a progestin-receptor agonist and suppresses the proliferation of cancer cells in ER+ breast cancer [29]. To further elucidate the therapeutic potential of these candidate drugs, we tested the expression levels of target genes of the four candidate drugs between breast cancer and normal samples, and they exhibited contrasting expression differences (Figure 3). This suggests that these drugs may intervene in the disease process by regulating target expression and may become meaningful treatment options."

Page 15, section "Case study: lung cancer" in the main manuscript.
"Alvocidib and docetaxel are in phase II clinical trials for the treatment of non-small cells [38]. We also tested the expression levels of target genes of the seven candidate drugs between lung cancer and normal samples, and they exhibited contrasting expression differences (Supplementary Figure S2)."

Point 3: The authors calculated centrality scores for disease candidate drugs by the random walk with restart algorithm, but the authors did not specify how the initial seed nodes were chosen. Different seed nodes would produce completely different results. And please append the stopping condition for random walk with restart algorithm.

Response 3: Thank you for your comments. We indeed used the random walk with restart algorithm to calculate the centrality scores of drugs. However, in our study, we applied this algorithm to a weighted

drug-drug functional similarity network, where the weights adopt the functional similarity between drugs (based on GO terms) in the context of the effect of disease on transcriptionally altered. The weighted edges reflect the evidence that a drug(s) may play an important latent role can be expected to be reinforced by the evidence of its neighbors. This observation suggests an approach based on random walks as a natural way of numerically assigning scores to drugs that reflect this notion of importance. Indeed, our network was constructed specifically with this idea in mind. By row-normalizing the adjacency matrix of the weighted network, we obtained a probability transition matrix for a random walk, where each element is the probability that starting at a drug node, the next step will be to another drug node. The weights on the edges in our network, reflected in the adjacency matrix, will bias the random walk in such a way that the walk proceeds along thicker (heavier) edges with greater probability than thinner (lighter) ones (Step 4, Figure 1).

The particular random walk with restart algorithm that we used here is defined as the limiting distribution of a random walk, i.e., the relative frequency at which you would find a random walker at each of the nodes in the network, in the limit of walking for a very long time. As such, the starting seeds are not important. What is important is the use of random restarts, because if the network has a strong community structure, the random walking can be slower than one might hope as the walker stays in one community or another for a long time. The algorithm itself is based on the original Google PageRank algorithm, which was developed that way because the World Wide Web displays this type of strongly clustered substructure and needs the periodic restart to nudge the walker into other communities. Since that algorithm is run for a long time, the dependence on the initial seed nodes is lost. And thus the seed nodes are selected from a uniform distribution in our study. To do this, the initial probability vector is constructed by assigning to each node with the same value and making their sum to be 1.

To sum up, we applied the random walk with restart algorithm to the weighted drug-drug network in our particular case. It is different from the general random walk algorithm which is applied in a non-weight network, and whose result depends on the selection of seed nodes. In this study, the random walk concludes when the centrality difference between two consecutive iterations falls below 1e-10. We consider this to be a convergence of node centrality, providing the most accurate reflection of a drug's potential effect.

We have strengthened the descriptions of the random walk algorithm used in our study. The revisions are as follows.

Page 9-10, section “Prioritizing disease candidate drugs through a network propagation algorithm” in the main manuscript.

“where T is the probability transition matrix; r is the restart probability. It has been demonstrated to have only a slight effect on the results when it fluctuated between 0.1 and 0.9 [25], we set r = 0.9 in the study. v0 is the initial probability vector. The random walk with restart applied here is characterized as the limiting distribution resulting from a prolonged random walk. Due to the algorithm's protracted runtime, it eventually becomes independent of the initial seed nodes, rendering the selection of starting seeds inconsequential. What matters lies in the strategic implementation of random restarts, particularly in networks featuring a robust community structure. And thus the seed nodes are selected from a uniform distribution in our study. To do this, the initial probability vector is constructed by assigning to each node with the same value and making their sum to be 1.

The parameter vt is the probability vector comprising the probabilities of the nodes at step t; After a limited number of iterations, the probability vector vt will converge to a stable state v (the difference between vt and vt+1 is less than 1e-10). Its ith element vi in the vector represents the eigenvector centrality score of drug Di. A larger eigenvector centrality score of a drug suggests a potential therapeutic effect on the disease.”

Point 4: To assess the robustness of the DrugSim2DR approach, the authors removed gene expression values from 5% to 20% at 5% intervals, but the tests were only repeated 20 times for each percentage of removal, which was unconvincing. It is suggested that the authors could increase the number of experiments to validate the method's robustness.

Response 4: Thank you very much for your suggestions. Following your advice, we increased the number of repetitions for each data removal experiment to 50 times. We obtained results similar to the previous ones, indicating that different data removal has only a minor impact on prediction accuracy. We have revised the relevant descriptions in the main manuscript and updated Figure 5B and Supplementary Figure

S3B to show the results.

Page 17, section "Robustness analysis of DrugSim2DR" in the main manuscript.
"To further assess the robustness of the DrugSim2DR, we performed the data removal tests using the BRC and LUA datasets. We removed the gene expression values from 5% to 20% at 5% intervals and reapplied the DrugSim2DR approach 50 times for each removal. For each removal, we performed the ROC analysis and calculated the AUROC values. The results showed that the different data removals had only a weak effect on the accuracy of the predictions. The median AUROC value remained above 0.75, even after the removal of up to 20% of the gene expression data in the BRC and LUA datasets (Figure 5B and Supplementary Figure S3B), indicating that the DrugSim2DR approach is robust to data removal."

Minor comments

Point 1: Please indicate whether disease and control samples were paired in the expression data used in the manuscript.

Response 1: Thank you very much for your suggestions. In this study, each set of gene expression data used contained non-paired disease and control samples. We have added relevant descriptions in the main manuscript.

Page 6, section "Data acquisition and processing" in the main manuscript.
"The transcriptional data of different cancer types were downloaded from the GEO database (https://www.ncbi.nlm.nih.gov/geo/), including breast cancer (GSE53752[12], GSE42568[13], and GSE21422[14]), lung adenocarcinoma (GSE68465[15], GSE31210[16], and GSE74706[17]), T-cell prolymphocytic leukemia (GSE5788[18]), renal cell carcinoma (GSE53757[19]) and head and neck squamous carcinoma (GSE6631[20]). Each set of gene expression data used contained non-paired disease and control samples. In these datasets, the expression values for each gene were standardized to a normal distribution using the z-score normalization method across all samples."

Point 2: There are some detail issues in the manuscript that need to be rechecked and revised by the authors, for example, in the first sentence of the "Background", the capitalization of the first letter of the middle word is illogical.

Response 2: Thank you very much for your comments. We sincerely apologize for this oversight. Subsequently, we conducted a comprehensive review of the entire main manuscript and rectified any inappropriate expressions.

Page 3, section "Background" in the main manuscript.
"Cancer is one of the most common complex diseases in terms of morbidity and mortality, because of the complexity and diversity of cancer, finding effective therapeutic drugs for cancer patients remains a formidable challenge."

Page 10, section "Prioritizing disease candidate drugs through a network propagation algorithm" in the main manuscript.
"The drug centrality score was calculated in the drug similarity network, which was constructed based on the intersection of each pair of drug target gene sets and GO terms and their transcriptional dysregulation scores, DE-scores."

Point 3: When estimating the transcriptional dysregulation level of genes, the authors should specify the details of the t-test, such as whether it was a one-tailed or two-tailed test.

Response 3: Thank you very much for your comments. In this study, we utilized a two-tailed t-test to calculate differential expression scores for genes. We have provided additional explanations regarding the t-test in the main manuscript.

Page 6, section "Calculating transcriptional dysregulation levels of genes" in the main manuscript.
"We estimated the transcriptional dysregulation level of genes in the context of a specific disease (Step 1 in

Figure 1). For each gene, the two-tailed t-test was applied to assess the gene differential expression extent between normal and disease samples.”

Point 4: Some references should be included: Yang, F., Zhang, Q., Ji, X. et al. Machine Learning Applications in Drug Repurposing. Interdiscip Sci Comput Life Sci 14, 15-21 (2022). https://doi.org/10.1007/s12539-021-00487-8; Zhang, Y., Jiang, Z., Chen, C. et al. DeepStack-DTIs: Predicting Drug-Target Interactions Using LightGBM Feature Selection and Deep-Stacked Ensemble Classifier. Interdiscip Sci Comput Life Sci 14, 311-330 (2022). https://doi.org/10.1007/s12539-021-00488-;

Response 4: Thank you very much for your comments. We have cited the relevant references accordingly.

Page 23, section “References” in the main manuscript.
“2. Yang F, Zhang Q, Ji X, Zhang Y, Li W, Peng S, et al. Machine Learning Applications in Drug Repurposing. Interdiscip Sci. 2022;14 1:15-21. doi:10.1007/s12539-021-00487-8.”

“42. Zhang Y, Jiang Z, Chen C, Wei Q, Gu H and Yu B. DeepStack-DTIs: Predicting Drug-Target Interactions Using LightGBM Feature Selection and Deep-Stacked Ensemble Classifier. Interdiscip Sci. 2022;14 2:311-30. doi:10.1007/s12539-021-00488-7.”
