# Supplementary material for: DrugSim2DR: systematic prediction of drug functional similarities in the context of specific disease for drug repurposing
Source: Gigascience. 2023 Dec 19;12:giad104. doi: 10.1093/gigascience/giad104 (PMC10729734; doi:10.1093/gigascience/giad104)
Supplement: giad104_Reviewer_1_Report_Original_Submission — Xiaochen Bo -- 9/26/2023 Reviewed [file giad104_reviewer_1_report_original_submission.pdf]

Reviewer Report

Title: DrugSim2DR: Systematic prediction of drug functional similarities in the context of specific disease for drug repurposing

Version: Original Submission **Date:** 9/26/2023

Reviewer name: Xiaochen Bo

Reviewer Comments to Author:

Comments to the AuthorIn this paper, the authors developed a novel software package, named DrugSim2DR, to prioritize cancer-candidate drugs based on a weighted drug-drug functional similarity network constructed with the transcriptionally altered genes in the context of the specific disease state. They applied the method to different cancer types to show the predictive power and compare their results with other methods. I tried their software package and found it usable. Totally, this is a potentially interesting and publishable study. However, I have several concerns about this study.1.The author chose drugs for network construction based on the count of target genes, with a range from 3 to 500. Might this result in excessively high degrees in certain nodes, thereby introducing bias into the network?2.In this study, the authors employed molecular function from Gene Ontology as an intermediary for evaluating drug-drug similarity. Why wasn't the biological process selected instead?3.When evaluating drug repositioning performance compared to other methodologies, it is advisable to incorporate more detailed result comparisons. For instance, comparing the top ten candidate drugs identified by various methods, beyond solely relying on AUROC comparisons, can provide a more comprehensive assessment.4.The author highlights method reproducibility by comparing the top 50 drugs across diverse datasets. However, I recommend extending the assessment of result consistency beyond the top-ranked drugs and including correlation analysis for a comprehensive view of drug optimization list consistency.5.In the section titled "Robustness Analysis of DrugSim2DR," the validation of the method's robustness and reproducibility is conducted. I recommend that the author dedicate a separate paragraph to the aspects related to reproducibility to enhance the clarity of the article's structure.6.There are some grammatical problems in the passage. For example, the "statistical significance" in the part "Prioritizing disease candidate drugs through a network propagation algorithm" should be changed to "statistically significant". Please check the description of inappropriate parts of the text and correct it.7.I suggest using "where" to link equations (2) and (3), or alternatively, swapping their order to enhance reader comprehension.

Level of Interest

Please indicate how interesting you found the manuscript: Choose an item.

Quality of Written English

Please indicate the quality of language in the manuscript: Choose an item.

Declaration of Competing Interests

Please complete a declaration of competing interests, considering the following questions:

- Have you in the past five years received reimbursements, fees, funding, or salary from an organisation that may in any way gain or lose financially from the publication of this manuscript, either now or in the future?
- Do you hold any stocks or shares in an organisation that may in any way gain or lose financially from the publication of this manuscript, either now or in the future?
- Do you hold or are you currently applying for any patents relating to the content of the manuscript?
- Have you received reimbursements, fees, funding, or salary from an organization that holds or has applied for patents relating to the content of the manuscript?
- Do you have any other financial competing interests?
- Do you have any non-financial competing interests in relation to this paper?

If you can answer no to all of the above, write 'I declare that I have no competing interests' below. If your reply is yes to any, please give details below.

I declare that I have no competing interests.

I agree to the open peer review policy of the journal. I understand that my name will be included on my report to the authors and, if the manuscript is accepted for publication, my named report including any attachments I upload will be posted on the website along with the authors' responses. I agree for my report to be made available under an Open Access Creative Commons CC-BY license (http://creativecommons.org/licenses/by/4.0/). I understand that any comments which I do not wish to be included in my named report can be included as confidential comments to the editors, which will not be published.

Choose an item.

To further support our reviewers, we have joined with Publons, where you can gain additional credit to further highlight your hard work (see: https://publons.com/journal/530/gigascience). On publication of this paper, your review will be automatically added to Publons, you can then choose whether or not to claim your Publons credit. I understand this statement.

Yes Choose an item.
