# Supplementary material for: DrugSim2DR: systematic prediction of drug functional similarities in the context of specific disease for drug repurposing
Source: Gigascience. 2023 Dec 19;12:giad104. doi: 10.1093/gigascience/giad104 (PMC10729734; doi:10.1093/gigascience/giad104)
Supplement: giad104_Reviewer_2_Report_Original_Submission — Dongqing Wei -- 10/6/2023 Reviewed [file giad104_reviewer_2_report_original_submission.pdf]

Reviewer Report

Title: DrugSim2DR: Systematic prediction of drug functional similarities in the context of specific disease for drug repurposing

Version: Original Submission **Date:** 10/6/2023

Reviewer name: Dongqing Wei

Reviewer Comments to Author:

Comment to the authorsThe authors proposed a novel algorithm named as DrugSim2DR, which could identify candidate anti-cancer drugs. They used common biological functions as mediators to construct a drug-drug functional similarity network in the specific disease transcriptome data to identify candidate anti-cancer drugs. They then applied DrugSim2DR to breast and lung cancer datasets and achieved good predictive performance. Moreover, DrugSim2DR also showed higher AUROC values by comparing it with other methods. Thus, DrugSim2DR may complement the existing drug repurposing methods. Overall, the manuscript is publishable. However, some issues need to be revised before it published.Major:1. In the data processing section, the authors described the drug selection process predicated on the count of target genes. I deem this preliminary processing to be essential. However, I did not come across an account of how the authors handled molecular function data. If the authors refrained from preprocessing it, kindly elucidate the rationale. Alternatively, kindly specify the strategy employed for processing.2. In the breast cancer case study, the authors identified five potential anti-breast cancer drugs, but only four of them are shown in Figure 3. I think the authors should make a reasonable explanation. The same problem appeared in the lung cancer case.3. The authors calculated centrality scores for disease candidate drugs by the random walk with restart algorithm, but they did not specify how the initial seed nodes were chosen. Different seed nodes may produce different results. Please explain this issue.4. To assess the robustness of the DrugSim2DR approach, the authors removed gene expression values from 5% to 20% at 5% intervals, but the tests were only repeated 20 times for each percentage of removal. It is suggested that the authors could increase the number of experiments to validate the method's robustness.Minor:1. Please indicate whether disease and control samples were paired in the expression data used in the manuscript.2. There are some detail issues in the manuscript that need to be rechecked and revised by the authors, for example, in the first sentence of the "Background", the capitalization of the first letter of the middle word is illogical.3. When estimating the transcriptional dysregulation level of genes, the authors should specify the details of the t-test, such as whether it was a one-tailed or two-tailed test.Some references should be included: Yang, F., Zhang, Q., Ji, X. et al. Machine Learning Applications in Drug Repurposing. Interdiscip Sci Comput Life Sci 14, 15-21 (2022). https://doi.org/10.1007/s12539-021-00487-8; Zhang, Y., Jiang, Z., Chen, C. et al. DeepStack-DTIs: Predicting Drug-Target Interactions Using LightGBM Feature Selection and Deep-Stacked Ensemble Classifier. Interdiscip Sci Comput Life Sci 14, 311-330 (2022). https://doi.org/10.1007/s12539-021-00488-;

Level of Interest

Please indicate how interesting you found the manuscript: Choose an item.

Quality of Written English

Please indicate the quality of language in the manuscript: Choose an item.

Declaration of Competing Interests

Please complete a declaration of competing interests, considering the following questions:

- Have you in the past five years received reimbursements, fees, funding, or salary from an organisation that may in any way gain or lose financially from the publication of this manuscript, either now or in the future?
- Do you hold any stocks or shares in an organisation that may in any way gain or lose financially from the publication of this manuscript, either now or in the future?
- Do you hold or are you currently applying for any patents relating to the content of the manuscript?
- Have you received reimbursements, fees, funding, or salary from an organization that holds or has applied for patents relating to the content of the manuscript?
- Do you have any other financial competing interests?
- Do you have any non-financial competing interests in relation to this paper?

If you can answer no to all of the above, write 'I declare that I have no competing interests' below. If your reply is yes to any, please give details below.

I declare that I have no competing interests

I agree to the open peer review policy of the journal. I understand that my name will be included on my report to the authors and, if the manuscript is accepted for publication, my named report including any attachments I upload will be posted on the website along with the authors' responses. I agree for my report to be made available under an Open Access Creative Commons CC-BY license (http://creativecommons.org/licenses/by/4.0/). I understand that any comments which I do not wish to be included in my named report can be included as confidential comments to the editors, which will not be published.

Choose an item.

To further support our reviewers, we have joined with Publons, where you can gain additional credit to further highlight your hard work (see: https://publons.com/journal/530/gigascience). On publication of this paper, your review will be automatically added to Publons, you can then choose whether or not to claim your Publons credit. I understand this statement.

Yes Choose an item.
